# A Unified Local Risk Map for Uncertainty-Aware Mobile Robot Navigation in Cluttered and Dynamic Environments

**DOI:** 10.3390/s26123900

**Published:** 2026-06-19

**Authors:** Elena Stracca, Olga Napolitano, Lucia Pallottino, Paolo Salaris

**Affiliations:** 1 Centro di Ricerca “E. Piaggio”, Dipartimento di Ingegneria dell’Informazione, Università di Pisa, Largo L. Lazzarino 1, 56122 Pisa, Italy; lucia.pallottino@unipi.it (L.P.); paolo.salaris@unipi.it (P.S.); 2 Faculty of Science, Engineering and Computing, Department of Electrical, Electronic and Robotic Engineering, Kingston University, Roehampton Vale, London SW15 3DW, UK; o.napolitano@kingston.ac.uk

**Keywords:** uncertainty-aware planning, risk-aware navigation, mobile robotics

## Abstract

Achieving safe and efficient navigation in cluttered and dynamic environments remains an open challenge for mobile robots, especially when perception and actuation are uncertain. Standard navigation stacks typically handle obstacle avoidance through fixed safety margins or costmap inflation layers. While effective in simple settings, these approaches are difficult to tune in practice: conservative inflation can prevent traversal through narrow passages, whereas less conservative settings may lead to unsafe behavior. Moreover, they usually encode risk only as a function of obstacle proximity. We propose a unified probability-inspired risk-cost map that integrates perception uncertainty, actuation uncertainty, dynamic obstacle prediction, and occlusion-aware memory into a single spatial representation. The resulting risk map is used by a local path-modification module that adapts a reference global path using the proposed risk map and interfaces with a standard Model Predictive Path Integral (MPPI) controller. The proposed method is compatible with standard navigation pipelines. We validate the resulting framework in Gazebo simulations under different sensing and actuation uncertainty conditions and in environments containing unknown static and dynamic obstacles. The results show that the proposed method is more robust than conventional costmap-based baselines, resulting in fewer aborted goals in cluttered environments and substantially fewer collision events when dynamic obstacles are present.

## 1. Introduction

Safety and performance are essential for autonomous mobile robots operating in environments shared with humans. In these scenarios, even a minor navigation error can lead to collisions with people or equipment. Traditional navigation systems maintain a predefined safety margin around the robot or obstacles [1], inflating obstacles by a fixed radius to keep a minimum distance. Extensions of this idea enforce fixed clearance directly at the planning level, for instance by encoding constant configuration-space buffers that guarantee collision-free tracking under model uncertainty [2]. However, large margins make the robot overly conservative, whereas small margins increase collision risk when unexpected hazards appear. In practice, industrial systems often tune inflation layers and local planners to balance safety and efficiency in specific environments such as warehouses [3], but these rules do not fully capture the spectrum of uncertainties and dynamic factors present in real-world scenarios.

To address these limitations, a prominent line of work formulates motion planning as a chance-constrained optimization problem, ensuring that the probability of collision along a trajectory remains below a given threshold [4,5]. Related approaches adopt risk measures such as Conditional Value-at-Risk to penalize rare but high-impact collision events, enabling planners to remain cautious in high-risk situations without being conservative [6]. These frameworks are powerful, but they typically model uncertainty only in terms of robot and obstacle states and do not capture the broader contextual and perceptual factors that affect safety.

Multiple sources of uncertainty must, therefore, be considered. On the perception side, limited field of view, occlusions, and sensing noise create regions where obstacles or humans may be present but undetected. Recent work has derived formal safety guarantees under imperfect observations [7] and developed occlusion-aware planners that treat hidden agents in a worst-case or probabilistic manner [8,9]. These methods highlight that unsafe behavior can arise not only from inaccurate state estimates, but also from unobserved areas that are implicitly treated as free. However, they often require specialized planning frameworks, which can make their integration into standard costmap-based navigation stacks less straightforward or impossible.

Beyond perception, dynamic agents introduce an additional dimension of risk. Humans and other moving entities behave stochastically and may change direction abruptly, especially in crowded environments. Modern approaches couple human motion prediction with risk-sensitive planning and control. For example, multimodal predictors integrated into stochastic MPC allow robots to trade off efficiency and caution via tunable risk parameters [10], while risk-biased forecasting and distributionally robust safety filters focus explicitly on rare but safety-critical behaviors [11,12,13]. These methods improve safety in dense, uncertain crowds, but typically do not provide a unified, spatially continuous view of risk that is instead needed by standard navigation stacks.

### Contributions

This work addresses the limitations of conventional obstacle inflation-based navigation by introducing a local risk-aware framework that explicitly accounts for uncertainty in perception and robot motion. Rather than relying solely on geometric clearance, the proposed approach constructs a unified, spatial representation of collision-related risk, which can be directly integrated into standard costmap-based navigation pipelines.

The primary contribution of this paper is the design of a unified local risk map that combines multiple sources of uncertainty and contextual risk into a single, continuous representation suitable for real-time planning. Specifically, the proposed map integrates perception uncertainty, actuation-induced motion uncertainty, dynamic obstacle predictions, and occlusion-aware memory within a common spatial framework, while also allowing the inclusion of offline-defined risk areas.

The other components of the framework are designed to support the construction and use of this representation. In particular:

- Uncertainty Propagation Pipeline: We develop a lightweight method to estimate obstacle detection uncertainty from LiDAR data and robot actuation uncertainty from tracking residuals, and propagate these quantities to the risk map.
- Risk-Aware Local Path Adaptation: We introduce a local replanning module based on A* search that operates on the unified risk map to adapt the reference global path to the current environment. In cluttered or dynamic settings where the global plan may traverse densely obstructed regions, this module modifies a local prefix of the path before passing it to the downstream controller. The same risk map also replaces the standard obstacle-inflation costmap used by the controller for trajectory evaluation, so that both the reference path and the control-level cost reflect the current uncertainty-aware risk assessment.
- Experimental Validation: We evaluate the proposed representation in simulation under varying uncertainty conditions and dynamic environments, demonstrating improved robustness compared to conventional costmap-based approaches.

## 2. Related Work

Safe navigation in dynamic partially unstructured environments has been addressed through several methodologies, including fixed safety margins, probabilistic risk assessment, uncertainty-aware perception, dynamic agent modeling, and context-dependent risk encoding.

### 2.1. Fixed Safety Margins and Obstacle Inflation

Classical navigation pipelines ensure safety by inflating obstacles according to the robot’s footprint and a fixed safety margin [1]. These margins can be enforced directly at the planning level, as in recent multi-robot frameworks that impose constant configuration-space buffers to guarantee collision-free tracking under model uncertainty [2]. Although effective, fixed clearance becomes overly conservative in dense or dynamic settings.

In human-shared environments, inflation is often interpreted as enforcing personal space. Planners treat each person like a larger obstacle with a fixed radius, which discourages robots from getting too close. Examples include Social Force–based navigation with collision-prediction elements [14] and hierarchical multi-agent planners that maintain non-overlapping safety zones [15].

Inflation also appears at the perception layer, where traversability and costmap inflation increase robustness to sensing uncertainty or complex terrain [3,16]. However, uniform inflation is limited: it ignores human motion, directionality, and task context. Consequently, several works propose asymmetric or dynamic inflation regions that account for pedestrian motion direction or speed, reducing unnecessary conservatism and improving interaction quality [17,18,19].

Overall, obstacle inflation provides strong safety guarantees but struggles to scale when the required margin varies with context, intent, or environmental risk.

### 2.2. Risk-Aware Navigation via Collision Probability

Risk-aware methods go beyond fixed safety margins by explicitly modeling the probability of collision. They build uncertainty into the planning process, which is especially helpful in dynamic environments with people [20].

Chance-constrained planners limit the collision probability along a trajectory [4]. Extensions include motion-planning methods based on linear–quadratic–Gaussian control [5] and model predictive control (MPC) formulations that add probabilistic safety constraints without simplifying the system dynamics to a linear model [21]. Other work uses random sampling (Monte Carlo techniques) to estimate the combined collision risk with multiple pedestrians [22], or applies Conditional Value-at-Risk (CVaR) to penalize rare but severe events and encourage conservative behavior in highly uncertain settings [6].

Risk can also be handled through reachability analysis, where forward-reachable sets together with analytical approximations of risk are used to discard unsafe trajectories in real time [23]. Extensions of barrier-function methods turn chance constraints into direct control constraints, enabling multi-robot coordination under uncertainty [24]. Beyond trajectory planning, chance-constrained formulations of Partial Observable Markov Decision Processes (POMDPs) treat risk as an explicit constraint, allowing policies that optimize performance while still enforcing probabilistic safety [25].

Earlier work by Fulgenzi et al. [20] combined probabilistic occupancy grids with obstacle tracking and a risk-guided RRT planner, integrating occlusions and velocity estimation into the risk computation. Patil et al. [26] proposed an analytical method to estimate collision probability under Gaussian motion and sensing uncertainty, providing conservative bounds suitable for online planning. In an alternative direction, the Lambda-Field framework [27] replaces the Bayesian occupancy grid with a continuous counting-process representation specifically designed for risk assessment along paths; its dynamic extension [28] handles moving obstacles.

A common trait of many classical uncertainty-aware planning approaches is that key uncertainty parameters, such as sensing noise, process noise, or obstacle-state covariance, are assumed known a priori or calibrated offline. Under this assumption, the resulting risk profile can adapt to geometry and predicted motion, but it does not reflect changes in the actual quality of perception or actuation during execution. In this sense, these methods remain fundamentally different from the exponential inflation layers used in standard navigation stacks, yet their practical conservatism is still fixed by offline tuning. The main added value of online uncertainty estimation is that the spatial risk profile can adapt at runtime to the current sensing and tracking conditions, rather than being determined solely by static design parameters.

### 2.3. Perception Uncertainty and Occlusion Handling

Robots operating in human environments face partial observability, where occlusions and sensing limits hide potential hazards [29,30,31]. Recent work incorporates perception uncertainty directly into planning, enabling safety without excessive conservatism.

Some methods provide formal safety guarantees under uncertain observations, deriving sufficient conditions for maintaining long-horizon safety despite imperfect sensing [7]. Others model occlusions as worst-case adversarial interactions, generating collision-free trajectories even when obstacles remain hidden [8], or construct predictive risk fields that account for agents that may be occluded [32].

Beyond worst-case reasoning, human behavior can act as an implicit sensing signal: by interpreting pedestrian reactions, robots infer the potential presence of hidden agents [33]. Occlusion-aware MPC embeds reachable sets of unobserved agents into real-time constraints, ensuring safety in cluttered indoor environments [9]. More recent frameworks combine POMDPs, prediction models, and uncertainty estimation to create dynamic probabilistic safety shields that remain effective in dense crowds [34]. Other MPC-based approaches generate multiple locally optimal, risk-aware trajectories to achieve smooth, safe motion under occlusion [35].

Collectively, these methods highlight the importance of explicitly modeling unobserved space, although many are formulated within specialized planning or control frameworks rather than as reusable costmap-compatible spatial representations.

### 2.4. Modeling Dynamic Agents and Rare-Event Risk

Human motion is stochastic, multimodal, and includes safety-critical behaviors [36,37,38]. Modern approaches, therefore, combine prediction with risk-sensitive control or explicitly bias models toward tail events.

Multimodal predictors integrated into stochastic MPC allow robots to trade off efficiency and caution via adjustable risk parameters [10]. Other works bias the predictors themselves toward rare collision-inducing modes, improving downstream safety even with limited samples [11].

Human motion may also include intentional interactions, such as blocking. Classifiers trained to detect adversarial intent enable proactive avoidance strategies [39]. Risk-sensitive filters enforce safety despite distribution shift by constructing ambiguity sets around observed human motions [12], while adaptive CVaR barrier functions expand safety constraints only when necessary [13].

Finally, some approaches leverage humans as navigational cues: by identifying suitable leaders, robots reduce prediction effort, achieving safe and efficient motion in crowds [40].

These works capture rich human–robot interaction patterns but typically do not unify risk into a costmap-compatible spatial representation.

### 2.5. Context-Dependent and Heterogeneous Risk Modeling

Risk in human environments is heterogeneous: the consequences of contact vary with object type, semantics, and task context [41,42]. Recent work, therefore, embeds semantic and contextual information directly into risk representations.

Some methods augment costmaps with semantic risk layers, using RGB-D perception to classify obstacles and inflate regions according to risk category [43]. Others build semantic graphs with risk propagation, enabling anticipation of hidden or unlabeled hazards [44]. Language models have also been used to convert textual hazard descriptions into semantic costmaps, enabling zero-shot risk-aware navigation [45].

Risk-aware MPC formulations integrate context-dependent risk with prediction and CVaR constraints for robust navigation in dynamic crowds [46]. Learning-based policies also incorporate explicit collision-risk functions [47] or soft risk maps that modulate avoidance behavior in a graded manner [48].

Large-scale deployments such as campus-wide navigation trials further highlight the need for robust, context-aware risk modeling on real systems [49].

Table 1 summarizes how representative works from the literature relate to the proposed framework along key capability dimensions. Prior research has established the importance of probabilistic reasoning under uncertainty, explicit prediction of dynamic agents, occlusion-aware safety, and semantic modulation of navigation cost. However, these capabilities are often addressed in isolation or embedded within specialized planners and controllers rather than unified into a single reusable spatial representation. In contrast, this work proposes a probability-inspired risk-costmap framework that combines online estimation of perception and actuation uncertainty with dynamic obstacle prediction, occlusion-aware risk accumulation, and heterogeneous task-dependent risk layers in a common grid representation. This representation is then used consistently by both the local path adaptation module and the MPPI controller. As a result, the robot can adapt its effective safety margins online according to the current quality of sensing, tracking, and environmental dynamics, while preserving the interpretability and implementation convenience of a map-based navigation pipeline.

### 2.6. Proposed Framework

**Figure 1:**
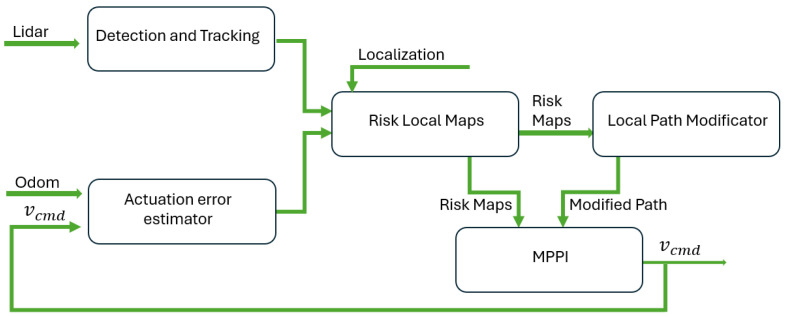
Block diagram of the proposed risk-aware navigation framework. The *Detection and Tracking* module processes raw LiDAR scans into circles and segments obstacles with per-component uncertainty estimates, while the *Actuation error estimator* converts the mismatch between commanded velocity $v_{cmd}$ and odometry into an actuation-uncertainty proxy. The *Risk Local Maps* block fuses these uncertainties together with localization information, dynamic-obstacle prediction, occlusion-aware memory, and offline-defined risk areas into a unified local risk-cost map. The resulting risk maps are consumed by two downstream blocks: the *Local Path Modifier* performs A* prefix replanning to adapt the global path locally, and the MPPI controller uses the same risk map (in place of the standard inflation costmap) together with the modified reference path to produce the velocity command $v_{cmd}$.

Building on the above literature, the proposed approach treats local navigation risk as a multi-layered spatial representation in which collision-related uncertainty, occlusion-induced uncertainty, dynamic prediction, and task-dependent contextual risk are encoded in a common map structure. As illustrated in Figure 1, the framework combines online uncertainty estimation, local risk-map construction, and risk-aware planning within a single pipeline. Collision-related risks arising from perception uncertainty, actuation noise, obstacle motion, and occlusions are encoded in a probabilistic collision-risk map, providing a spatially continuous estimate of collision-related likelihood. In particular, the map integrates uncertainty in obstacle detection and tracking, the estimated positional spread induced by robot actuation uncertainty, and the predicted motion of dynamic obstacles over a finite horizon. Visibility reasoning is additionally used to preserve previously observed collision risk only in regions that are currently occluded, preventing recently hidden obstacles from being immediately treated as free space.

Task-level and semantic risks are represented through dedicated risk-area maps, which encode restricted zones and regions associated with increased operational cost. These areas may include, for example, regions near emergency exits or zones where undetectable or partially observable objects may be present. In the proposed framework, such risk areas and their associated severity are assumed to be assessed offline or updated online when available and treated as known priors. Since the presence of the robot within a restricted area is not, in general, directly observable, the contribution of these risk factors is evaluated jointly with the robot’s current localization uncertainty. The associated risk, therefore, reflects not only the spatial extent and severity of the area but also a measure of the probability that the robot occupies it given its estimated pose. The resulting layers are combined into a local risk-cost representation used by the replanning module to navigate cluttered environments and are interfaced with the MPPI controller. Besides collision and restricted-area violations, the framework also addresses task-level failures. In cluttered environments, the safety mechanisms of the baseline controller often avoid collisions by stopping the robot when it gets too close to obstacles. However, this may result in aborted executions and, when the recovery behaviors are unable to restore a feasible configuration, in the robot becoming stuck and unable to complete the task. Table 2 summarizes the main risk factors addressed in this work and the associated framework components.

## 3. Detection and Actuation Uncertainty Estimation

This section describes how uncertainty quantities used by the local risk map are estimated online. The goal is not to obtain fully consistent probabilistic models, but to derive computationally efficient uncertainty proxies suitable for real-time collision-risk evaluation. In particular, obstacle state uncertainty is estimated from LiDAR detections, and robot actuation uncertainty from tracking residuals. These uncertainties are propagated to the local collision-risk map described in Section 4.

### 3.1. Obstacle Detection and Tracking

In this work, obstacle extraction and tracking are performed entirely from 2D LiDAR observations. Raw scans are clustered into two geometric primitives: *circles* (compact, possibly moving obstacles) and *segments* (known static obstacles such as walls). We build on the public obstacle_detector package (https://github.com/tysik/obstacle_detector (accessed on 15 June 2026)), which provides segmentation logic and a Kalman filter to track circle obstacles [50]. The original system focuses on tracking dynamic circles; we extend it to track all circle and segment obstacles, using constant-velocity Kalman filters for circles and Welford’s online algorithm for segments, with adaptive measurement-noise estimation for both primitive types. The main additions over the original implementation include: per-component measurement-noise estimation fusing innovation-based and detector-based uncertainty, a dedicated random-walk model for the radius, a dynamic/static classification scheme with hysteresis, variance inflation during detection gaps, and propagation of per-obstacle uncertainty to the collision-probability map described in Section 4.

#### 3.1.1. Circle Obstacles

Each detected compact obstacle not already known from the static map is approximated as a circle, parameterized by its center $c_{o}=\left(x_{o},y_{o}\right)$ and radius $r_{o}$. The state of a circle obstacle *j* is given by its center position and velocity, and its radius:

$$x_{o,j}={\left[\begin{array}{ccccc} x_{o,j} & y_{o,j} & v_{x,j} & v_{y,j} & r_{o,j} \end{array}\right]}^{\;⊤}.$$

The position components are estimated by two independent constant-velocity Kalman filters for $\left(x_{o},v_{x}\right)$ and $\left(y_{o},v_{y}\right)$, each with state dimension n=2 and measurement dimension m=1. Denoting by Δt the filter time step and by d∈{x,y} the position components, their state-transition and observation matrices are

$$A_{d}=\left[\begin{array}{cc} 1 & \Delta t\\ 0 & 1 \end{array}\right],\;H_{d}=\left[\begin{array}{cc} 1 & 0 \end{array}\right],$$

so that the predicted measurement is simply the position element of the predicted state. The process-noise covariance for each position component follows the piecewise-constant white-noise acceleration model,

$$Q_{d}=\sigma_{a}^{2}\left[\begin{array}{cc} \Delta t^{3}/3 & \Delta t^{2}/2\\ \Delta t^{2}/2 & \Delta t \end{array}\right],\;d\in \left\{x,y\right\},$$

where $\sigma_{a}^{2}$ is the acceleration power spectral density. In the original implementation [50], all three channels shared the same constant-velocity structure and identical diagonal process-noise entries. We instead model the radius with a separate scalar random-walk filter ($A_{r}=H_{r}=1$, $Q_{r}=10^{-6}\;\Delta t$), since the physical radius of a rigid obstacle is constant and does not require a velocity component.

The original tracker used a single adaptive measurement variance, estimated from the total squared position innovation and applied identically to all three channels. We replace this scheme with a per-component estimation that draws on two complementary sources and fuses them conservatively. Let ${\hat{x}}_{d,j,k}^{-}$ and $P_{d,j,k}^{-}$ denote the predicted state and covariance of the *d*-th filter for obstacle *j* at step *k*, and let $z_{o,j,k}={\left[x_{o,j,k},\;y_{o,j,k},\;r_{o,j,k}\right]}^{\;⊤}$ be the detector output. The innovation for each component d∈{x,y,r} is

$$\nu_{d,j,k}=z_{d,j,k}-H_{d}\;{\hat{x}}_{d,j,k}^{-}.$$

In a correctly tuned filter, the expected squared innovation equals the innovation covariance $S_{d,j,k}=H_{d}\;P_{d,j,k}^{-}\;H_{d}^{\;⊤}+R_{d,j,k}$. Rearranging for *R* and replacing the expectation with the single-sample realization $\nu_{d,j,k}^{2}$ gives an instantaneous innovation-based estimate

$${\hat{R}}_{d,j,k}=\max\;\left(\epsilon ,\;\nu_{d,j,k}^{2}\;-\;H_{d}\;P_{d,j,k}^{-}\;H_{d}^{\;⊤}\right),$$

where ϵ>0 is a small numerical floor. Because the estimate relies on a single innovation sample rather than its expectation, the argument of the max can occasionally become negative when a measurement happens to lie close to the prediction; the floor ϵ ensures that $\hat{R}$ remains a valid variance in those cases. A second, independent estimate, absent in the original implementation is obtained from the circle-fitting residuals produced by the detector. Let $n_{j,k}$ denote the number of LiDAR points associated with obstacle *j* at step *k*, and let $q_{i}$ be the *i*-th such point. To make the estimate reflect how much the circle boundary may underestimate the true obstacle extent, rather than how well the obstacle conforms to a circular shape, only points lying outside the fitted boundary contribute:

$$R_{j,k}^{\mathrm{ext}}=\max\;\left(\epsilon ,\;\frac{1}{n_{j,k}}\frac{\sum_{i=1}^{n_{j,k}}{\left[\max\;\left(0,\;∥q_{i}-c_{j}∥-r_{j}\right)\right]}^{2}}{\max(1,\;n_{j,k}-3)}\right),$$

where the three degrees of freedom are subtracted in the denominator account for the fitted circle parameters. The additional $1/n_{j,k}$ factor converts the Bessel-corrected sample variance into a variance of the mean, so that $R_{j,k}^{\mathrm{ext}}$ reflects the uncertainty of the boundary position estimate rather than the per-point scatter; consequently, well-observed obstacles (many points) receive tighter uncertainty bounds. Since this estimate depends only on the point cloud geometry, the same value is used for all three components. The two sources are fused by taking their maximum,

$$R_{d,j,k}^{\mathrm{tar}}=\max\;\left({\hat{R}}_{d,j,k},\;R_{j,k}^{\mathrm{ext}}\right),$$

so that the adopted noise is never smaller than either source suggests. To reduce sensitivity to outliers or brief occlusions, the target value is smoothed with an exponential moving average,

$$R_{d,j,k}=\alpha_{R,d}\;R_{d,j,k-1}+\left(1-\alpha_{R,d}\right)\;R_{d,j,k}^{\mathrm{tar}},$$

where $\alpha_{R,x}=\alpha_{R,y}$ and $\alpha_{R,r}$ are smoothing coefficients in [0,1] (a lower value allows faster adaptation at the cost of stability). The smoothed values, clipped to the interval $\left[\epsilon ,\;R_{\max}\right]$, form the diagonal of the measurement-covariance matrix used in the Kalman correction step. When a new obstacle track is created, its initial *R* is set to the running exponential average of the *R* values across all active tracks, so that new tracks inherit a representative noise level from the outset rather than an arbitrary fixed default.

A detected circle is not immediately promoted to a tracked obstacle: it must first be consistently matched to the detector output for a minimum number of consecutive frames. Once promoted, its initial velocity is bootstrapped from the displacement observed between the first and last staging observations. Each tracked obstacle is then classified as either staticor dynamic. An obstacle is considered dynamic only when two conditions are simultaneously satisfied: its estimated velocity is statistically significant, as assessed by a Mahalanobis-distance test on the velocity components, and it exhibits coherent net displacement over a sliding window. Requiring both criteria prevents sensor noise or momentary vibrations from triggering false dynamic classifications. A hysteresis counter governs the transitions: promotion to dynamic requires a short burst of positive evidence, while reversion to static demands that the counter drop to zero, avoiding spurious reclassifications during direction changes. When an obstacle transitions from static to dynamic, the position process noise is increased ($\sigma_{a,\mathrm{dyn}}^{2}=5\;\sigma_{a}^{2}$) so that the filter can follow rapid manoeuvres, without losing the obstacle track; conversely, confirmed static obstacles have their velocity estimate zeroed after a brief settling period to prevent drift.

For the obstacle centre, the uncertainty used downstream is taken directly from the a posteriori covariance of the Kalman filters, namely

$$\sigma_{o,x,j}^{2}=P_{x,x,j,k}^{+},\;\sigma_{o,y,j}^{2}=P_{y,y,j,k}^{+}.$$

For the radius, however, the random-walk model uses a deliberately very small process noise in order to prevent spurious temporal drift of the estimated obstacle size. As a consequence, the posterior covariance $P_{r,r,j,k}^{+}$ mainly reflects filter confidence under the assumed model and may underestimate the actual dispersion induced by sensing noise and imperfect circle fitting. To obtain a more conservative quantity for collision-risk evaluation, we therefore, augment the posterior radius variance with the current measurement-noise estimate and define the reported radius uncertainty as

$$\sigma_{o,r,j}^{2}=P_{r,r,j,k}^{+}+R_{r,j,k}.$$

This quantity should be interpreted as an effective uncertainty proxy used for downstream collision-probability computation, rather than as the exact posterior variance of a fully consistent Bayesian estimator. All five variance components ($\sigma_{o,x}^{2}$, $\sigma_{o,y}^{2}$, $\sigma_{o,r}^{2}$, $\sigma_{v_{x}}^{2}$, $\sigma_{v_{y}}^{2}$) are sent to the collision-probability map. To account for temporal detection coherence, if a tracked obstacle exists at time *t* it is expected nearby at t+1, when an obstacle is not matched to any detector output for δ consecutive frames, its predicted covariance is inflated as $P_{d,d}^{-}\leftarrow P_{d,d}^{-}+\gamma \;\delta$ for d∈{x,y}. This makes the system robust to momentary detection failures: the downstream collision-probability map becomes more conservative around these obstacles until the track is eventually deleted.

#### 3.1.2. Segment Obstacles

A segment is defined by its two endpoints $p^{f}={\left(p_{x}^{f},p_{y}^{f}\right)}^{⊤}$ and $p^{l}={\left(p_{x}^{l},p_{y}^{l}\right)}^{⊤}$, denoting the *first* and *last* point, respectively. For each tracked segment, we maintain the running mean $\bar{p}$ and a Welford accumulator $M_{2}$ for every endpoint coordinate, applying the update independently to each of the four scalar components $p_{x}^{f},\;p_{y}^{f},\;p_{x}^{l},\;p_{y}^{l}$. Given the statistics $\left({\bar{p}}_{n-1},\;M_{2,n-1}\right)$ after n−1 observations, the *n*-th measurement $p_{n}$ is incorporated as

$$\begin{array}{cc} \Delta_{n} & =p_{n}-{\bar{p}}_{n-1},\\ {\bar{p}}_{n} & ={\bar{p}}_{n-1}+\frac{1}{n}\;\Delta_{n},\\ M_{2,n} & =M_{2,n-1}+\Delta_{n}\left(p_{n}-{\bar{p}}_{n}\right), \end{array}$$

with $M_{2,0}=0$ and *n* denoting the cumulative observation count for the track. Cross-covariance between the *x* and *y* coordinates is neglected, yielding a diagonal covariance estimate. The procedure produces four accumulators $M_{2,x}^{f},\;M_{2,y}^{f},\;M_{2,x}^{l},\;M_{2,y}^{l}$, from which the Bessel-corrected sample variances follow as $\sigma_{e,a}^{2}=M_{2,a}^{e}/\left(n-1\right)$ for endpoint e∈{f,l} and coordinate a∈{x,y}.

After *n* observations, the mean segment direction and length are

$$d={\bar{p}}^{\;l}-{\bar{p}}^{\;f},\;L=∥d∥.$$

For a non-degenerate segment (L>0), the unit normal is $\hat{n}={\left({\hat{n}}_{x},\;{\hat{n}}_{y}\right)}^{⊤}={\left(-d_{y}/L,\;d_{x}/L\right)}^{⊤}$. Projecting the endpoint variances onto this direction gives the components relevant to point-to-segment distance:

$$\sigma_{f,⊥}^{2}={\hat{n}}_{x}^{2}\;\sigma_{f,x}^{2}+{\hat{n}}_{y}^{2}\;\sigma_{f,y}^{2},\;\sigma_{l,⊥}^{2}={\hat{n}}_{x}^{2}\;\sigma_{l,x}^{2}+{\hat{n}}_{y}^{2}\;\sigma_{l,y}^{2}.$$

The normal-direction variance at an arbitrary point parameterized by t∈[0,1] along the segment (t=0 at $p^{f}$, t=1 at $p^{l}$) is ${\left(1-t\right)}^{2}\;\sigma_{f,⊥}^{2}+t^{2}\;\sigma_{l,⊥}^{2}$, assuming endpoint independence. Since this expression is convex in *t*, its maximum over [0,1] is attained at one of the endpoints; we, therefore, adopt the conservative bound

$$\sigma_{s}^{2}=\max\;\left(\sigma_{f,⊥}^{2},\;\sigma_{l,⊥}^{2}\right)+q_{s}\;\delta_{s},$$

where $q_{s}$ is a per-frame process-noise parameter and $\delta_{s}$ is the number of consecutive frames in which the segment was not observed ($\delta_{s}=0$ when the segment is matched at the current step). The additive term plays the same role as the covariance inflation described for circles: it progressively increases the reported uncertainty for temporarily unobserved segments, making the downstream collision model more conservative until the track is eventually deleted. As with circles, a segment track must accumulate a minimum number of observations before being published, suppressing spurious detections.

For data association, at each iteration, the detected segments are matched to existing tracks using a geometric cost that combines center distance, distance in the normal direction, and angular alignment. Let $s_{\mathrm{new}}=\left(p_{\mathrm{new}}^{f},\;p_{\mathrm{new}}^{l}\right)$ and $s_{\mathrm{trk}}=\left({\bar{p}}_{\mathrm{trk}}^{\;f},\;{\bar{p}}_{\mathrm{trk}}^{\;l}\right)$ denote the new observation and the existing track mean, with segment medium point $m=\frac{1}{2}\left(p^{f}+p^{l}\right)$. The orthogonal distance is defined as $d_{⊥}=\frac{1}{2}\left(\right|r_{f}\left|+\right|r_{l}\left|\right)$, where $r_{f}={\hat{n}}_{\mathrm{trk}}^{⊤}\left(p_{\mathrm{new}}^{f}-{\bar{p}}_{\mathrm{trk}}^{\;f}\right)$ and $r_{l}={\hat{n}}_{\mathrm{trk}}^{⊤}\left(p_{\mathrm{new}}^{l}-{\bar{p}}_{\mathrm{trk}}^{\;f}\right)$ are the signed normal distances of the observed endpoints from the track line. Letting Δθ∈[−π,π] be the wrapped angular difference between the two segment orientations, the association cost is

$$c\left(s_{\mathrm{new}},\;s_{\mathrm{trk}}\right)=w_{m}\;∥m_{\mathrm{new}}-m_{\mathrm{trk}}∥+w_{⊥}\;d_{⊥}+w_{\theta }\;L_{\mathrm{trk}}\;\left|\Delta \theta \right|,$$

where $L_{\mathrm{trk}}$ is the track length and $w_{m},\;w_{⊥},\;w_{\theta }$ are tunable weights; the angular term is scaled by $L_{\mathrm{trk}}$ so that all three contributions are expressed in meters. A track is associated when

$$c\left(s_{\mathrm{new}},\;s_{\mathrm{trk}}\right)<\tau_{\mathrm{assoc}}+k_{\mathrm{gate}}\sqrt{\sigma_{s,\mathrm{trk}}^{2}},$$

where $\tau_{\mathrm{assoc}}$ is a base distance threshold and $k_{\mathrm{gate}}$ controls the gate expansion with the track’s current uncertainty $\sigma_{s,\mathrm{trk}}^{2}$. Unmatched tracks have their fade counter incremented and are deleted after a configurable timeout.

### 3.2. Actuation Uncertainty Estimation

The goal is to quantify the mismatch between the commanded motion and the motion effectively executed by the robot. This estimate is not intended to replace controller selection or low-level controller tuning. Instead, it accounts for residual and time-varying execution errors that may remain after tuning, for example, due to wheel slip, changing floor conditions, actuator limitations, imperfect modeling, payload changes, or external disturbances. This uncertainty is relevant for collision-risk estimation because it affects the spatial dispersion of the robot position over the local prediction horizon.

The robot state in the plane is denoted by $x_{r}={\left[x_{r},\;y_{r},\;\theta_{r}\right]}^{⊤}$. Assuming a differential-drive platform, the commanded input is $u_{r}={\left[v,\;\omega \right]}^{⊤}$, where *v* is the commanded linear velocity and ω is the commanded angular velocity. Actuation uncertainty is estimated online by comparing commanded velocities with odometry-based velocity measurements. Let $v_{\mathrm{cmd}}\left(t\right),\;\omega_{\mathrm{cmd}}\left(t\right)$ be the commanded inputs and $v_{\mathrm{odom}}\left(t\right),\;\omega_{\mathrm{odom}}\left(t\right)$ the odometry measured velocities. We define the tracking residuals as

$$e_{v}\left(t\right)=v_{\mathrm{odom}}\left(t\right)-v_{\mathrm{cmd}}\left(t\right),\;e_{\omega }\left(t\right)=\omega_{\mathrm{odom}}\left(t\right)-\omega_{\mathrm{cmd}}\left(t\right).$$

The estimator maintains sliding windows of the $N_{w}$ most recent residuals. The linear-velocity residual buffer is updated only when $|v_{\mathrm{cmd}}|$ exceeds a motion threshold, and the angular-velocity residual buffer only when $|\omega_{\mathrm{cmd}}|$ exceeds a turning threshold, so that standstill noise does not corrupt the estimates.

Given the buffered residuals ${\left\{e_{v}^{\left(i\right)}\right\}}_{i=1}^{N_{w}}$ (and analogously for $e_{\omega }$), the systematic tracking bias is estimated robustly as the sample median,

$${\bar{e}}_{v}=\mathrm{median}\;\left(\{e_{v}^{\left(i\right)}\}\right),$$

and the random dispersion is estimated through the median absolute deviation (MAD) [51],

$$\sigma_{v}=1.4826\;\mathrm{median}\;\left(\{|e_{v}^{\left(i\right)}-{\bar{e}}_{v}|\}\right),$$

where the factor 1.4826 makes the MAD consistent with the standard deviation for Gaussian data. The same procedure is applied independently to $\left\{e_{\omega }^{\left(i\right)}\right\}$, yielding ${\bar{e}}_{\omega }$ and $\sigma_{\omega }$.

To avoid unrealistically small uncertainty values when the buffers contain too little excitation or nearly constant samples, lower bounds $\sigma_{v,\min}$ and $\sigma_{\omega ,\min}$ are imposed on the estimated dispersions. In practice, these floors prevent the robot from becoming artificially overconfident during very slow motions or short stationary intervals.

To propagate actuation uncertainty to position space, we adopt a short-horizon first-order approximation. The goal is not to derive an exact stochastic motion model, but to obtain a compact uncertainty proxy suitable for collision-risk evaluation in the local planner. Over this horizon $T_{u}>0$, uncertainty in linear velocity induces a translational spread

$$\sigma_{\mathrm{trans}}=T_{u}\sigma_{v}.$$

Uncertainty in angular velocity instead produces a lateral displacement whose magnitude depends on the robot footprint. Using the nominal robot collision radius $r_{\mathrm{rob}}$ as a characteristic footprint scale, we approximate the positional effect of yaw-rate uncertainty by the equivalent lateral spread

$$\sigma_{\mathrm{ang}}=r_{\mathrm{rob}}\;T_{u}\sigma_{\omega }.$$

Neglecting the correlation between the linear and angular velocity residuals, we define the combined lateral dispersion as

$$\sigma_{\mathrm{lat}}=\sqrt{\sigma_{\mathrm{trans}}^{2}+\sigma_{\mathrm{ang}}^{2}}=T_{u}\sqrt{\sigma_{v}^{2}+r_{\mathrm{rob}}^{2}\sigma_{\omega }^{2}}.$$

The median residuals ${\bar{e}}_{v}$ and ${\bar{e}}_{\omega }$ represent instead systematic tracking biases rather than random dispersion. Under the same first-order approximation, we convert them into an equivalent lateral bias over horizon $T_{u}$:

$$b_{\mathrm{lat}}=T_{u}\left({\bar{e}}_{v}+r_{\mathrm{rob}}{\bar{e}}_{\omega }\right).$$

This term is not a variance contribution, but a conservative proxy for the mean displacement induced by persistent velocity and yaw-rate bias. Accordingly, we summarize the actuation-induced positional effect through a Gaussian surrogate with lateral mean shift $b_{\mathrm{lat}}$ and standard deviation $\sigma_{\mathrm{lat}}$. Since a single zero-mean scalar quantity is required by the downstream collision-risk map, we use the effective uncertainty proxy

$$\sigma_{r,\mathrm{eff}}=\sqrt{\sigma_{\mathrm{lat}}^{2}+{\left(\kappa |b_{\mathrm{lat}}|\right)}^{2}},$$

where κ∈[0,1] controls how strongly the systematic component is absorbed into the equivalent spread. This quantity is used for planning convenience and should not be interpreted as the exact variance of a probabilistic motion model. For collision-risk computation, the resulting positional uncertainty is finally approximated as isotropic in the local plane for computational simplicity, with 

$$\sigma_{r,x}=\sigma_{r,y}=\sigma_{r,\mathrm{eff}}.$$

## 4. Collision Risk Map

This section explains how the uncertainty estimates of Section 3 are converted into a spatial collision-risk map. The collision-risk map is a grid M in which each cell *c* stores a collision-related value associated with placing the robot center at $\left(x_{c},y_{c}\right)$. This value is computed from a measure of the probability that the robot, modeled as a disc of radius $r_{\mathrm{rob}}$, would overlap with at least one obstacle under uncertainty in both robot and obstacle states. The map explicitly accounts for robot actuation uncertainty (Section 3.2) and obstacle sensing uncertainty (Section 3.1), and is further complemented by visibility-based memory, dynamic-obstacle propagation, and footprint enlargement. However, it is not intended to be interpreted as a perfectly calibrated probability field. Indeed, several conservative approximations and planning-oriented heuristics are incorporated in the final construction in order to better capture practical safety requirements. Additional non-collision risk layers are introduced separately in Section 5.

Let $O_{s}$ and $O_{d}$ denote the sets of detected static and dynamic obstacles, respectively. For each obstacle *o*, we compute a per-cell collision probability $P_{\mathrm{coll}}\left(c|o\right)\in \left[0,1\right]$, defined as the probability that the uncertain robot footprint, when nominally centered at *c*, overlaps with the spatial extent of *o*. Under the simplifying assumption of conditional independence between obstacle-specific collision events, static and dynamic contributions are aggregated as

(1) $$\begin{array}{cc} P_{\mathrm{coll}}^{\mathrm{static}}\left(c\right)\; & =1-\prod_{o\in O_{s}}\left(1-P_{\mathrm{coll}}\left(c|o\right)\right),\\ P_{\mathrm{coll}}^{\mathrm{moving}}\left(c\right)\; & =1-\prod_{o\in O_{d}}\left(1-P_{\mathrm{coll}}\left(c|o\right)\right). \end{array}$$

### 4.1. Robot Positional Uncertainty

The collision-probability map and the local costmap operate in the odometry frame. Since obstacles are sensed via LiDAR, their positions are measured relative to the robot and are, therefore, expressed directly in the odometry frame, unaffected by drift between the odometry and map frames. For this reason, the robot positional uncertainty used in collision-probability computations accounts only for actuation uncertainty: localization uncertainty (e.g., from AMCL (Adaptive Monte Carlo Localization)) does not affect the relative robot–obstacle geometry in the odometry frame and is, therefore, excluded. Localization uncertainty is instead incorporated when evaluating offline-defined risk areas, which are specified in the map frame (see Section 5).

Rather than applying a uniform uncertainty over the whole map, we compute a cell-dependent estimate that scales with the distance of each cell from the robot, extending the fixed-horizon approximation introduced in Section 3.2. Let

$$d_{c}=∥(x_{c}-x_{r},\;y_{c}-y_{r})∥$$

be the Euclidean distance between the robot position and the center of cell *c*. For that cell, we define an effective prediction horizon

$$T_{\mathrm{eff}}\left(c\right)=\min\;\left(\frac{d_{c}}{\max(v_{\mathrm{rob}},\;\epsilon )},\;T_{u}\right),$$

where $v_{\mathrm{rob}}$ is the current robot speed, ϵ>0 is a small floor preventing division by zero, and $T_{u}$ is the maximum horizon introduced in Section 3.2.

Using the same first-order approximation adopted there, the translational contribution at cell *c* is

$$\sigma_{\mathrm{trans}}\left(c\right)=T_{\mathrm{eff}}\left(c\right)\;\sigma_{v},$$

while the angular contribution is converted into an equivalent lateral spread using the nominal robot collision radius $r_{\mathrm{rob}}$:

$$\sigma_{\mathrm{ang}}\left(c\right)=r_{\mathrm{rob}}\;T_{\mathrm{eff}}\left(c\right)\;\sigma_{\omega }.$$

Assuming independence between the two components, the actuation-induced random spread at cell *c* is

$$\sigma_{\mathrm{lat}}\left(c\right)=\sqrt{\sigma_{\mathrm{trans}}{\left(c\right)}^{2}+\sigma_{\mathrm{ang}}{\left(c\right)}^{2}}=T_{\mathrm{eff}}\left(c\right)\sqrt{\sigma_{v}^{2}+r_{\mathrm{rob}}^{2}\sigma_{\omega }^{2}}.$$

The same approximation is used to propagate the systematic residuals ${\bar{e}}_{v}$ and ${\bar{e}}_{\omega }$, yielding the equivalent lateral bias

$$b_{\mathrm{lat}}\left(c\right)=T_{\mathrm{eff}}\left(c\right)\left({\bar{e}}_{v}+r_{\mathrm{rob}}{\bar{e}}_{\omega }\right).$$

As in Section 3.2, this term is not interpreted as a variance contribution, but as a conservative proxy for the mean displacement induced by persistent actuation bias.

When a single scalar quantity is required by the downstream collision-probability model, we absorb this bias into an effective spread and define

(2) $$\sigma_{\mathrm{act}}\left(c\right)=\sqrt{\sigma_{\mathrm{lat}}{\left(c\right)}^{2}+{\left(\kappa |b_{\mathrm{lat}}\left(c\right)|\right)}^{2}},$$

where κ∈[0,1] controls how strongly the systematic component is converted into equivalent spread. This quantity is used as a planning-oriented uncertainty proxy rather than as the exact variance in a probabilistic motion model.

Finally, the robot positional uncertainty at cell *c* is approximated as isotropic in the local plane:

$$\sigma_{r,x}\left(c\right)=\sigma_{r,y}\left(c\right)=\max\;\left(\sigma_{\mathrm{act}}\left(c\right),\;\epsilon \right).$$

For cells close to the robot, $T_{\mathrm{eff}}\left(c\right)$ is small, and the resulting actuation uncertainty remains low. For sufficiently distant cells, $T_{\mathrm{eff}}\left(c\right)$ saturates at $T_{u}$, recovering the fixed-horizon estimate of Section 3.2.

### 4.2. Static Obstacles

Throughout this section, $r_{\mathrm{rob}}$ denotes the minimum robot collision radius, corresponding to the inscribed circle of the actual footprint, and Φ(·) denotes the standard normal cumulative distribution function.

#### 4.2.1. Circular Obstacles

Consider a static circular obstacle *o* with estimated center $c_{o}\in R^{2}$, radius $r_{o}$, position variances $\sigma_{o,x}^{2}$ and $\sigma_{o,y}^{2}$, and radius variance $\sigma_{o,r}^{2}$, all provided by the tracker of Section 3.1. Denoting by

$$d=∥(x_{c}-c_{o,x},\;y_{c}-c_{o,y})∥$$

the Euclidean distance between the cell center and the obstacle center, a collision occurs when $d\leq r_{\mathrm{rob}}+r_{o}$.

We approximate the collision-margin random variable $Y=D-(r_{\mathrm{rob}}+r_{o})$ as Gaussian, $Y∼N(\mu_{Y},\sigma_{Y}^{2})$, with 

$$\mu_{Y}=d-\left(r_{\mathrm{rob}}+r_{o}\right)$$

and conservative variance

$$\sigma_{Y}^{2}=\left(\sigma_{r,x}{\left(c\right)}^{2}+\sigma_{r,y}{\left(c\right)}^{2}\right)+\max\;\left(\sigma_{o,x}^{2},\sigma_{o,y}^{2}\right)+\sigma_{o,r}^{2}.$$

The first term accounts for robot actuation uncertainty (Section 4.1), the second for obstacle position uncertainty, using the worst-case axis for conservatism, and the third for radius uncertainty. The per-cell collision probability is then

(3) $$P_{\mathrm{coll}}\left(c|o\right)=\Phi \;\left(\frac{r_{\mathrm{rob}}+r_{o}-d}{\sigma_{Y}}\right).$$

#### 4.2.2. Segment Obstacles

For a segment obstacle with endpoints $\left(p^{f},p^{l}\right)$ and associated normal-direction variance $\sigma_{s}^{2}$ from Section 3.1, let *d* denote the Euclidean point-to-segment distance from $\left(x_{c},y_{c}\right)$ to the segment. A collision occurs when $d\leq r_{\mathrm{rob}}$. The combined uncertainty on the distance is

$$\sigma_{D}=\sqrt{\sigma_{r,x}{\left(c\right)}^{2}+\sigma_{r,y}{\left(c\right)}^{2}+\sigma_{s}^{2}},$$

and the per-cell collision probability follows the same CDF model:

$$P_{\mathrm{coll}}\left(c|s\right)=\Phi \;\left(\frac{r_{\mathrm{rob}}-d}{\sigma_{D}}\right).$$

### 4.3. Moving Obstacles and Uncertainty Propagation

For dynamic circular obstacles, we evaluate risk over a prediction horizon $T_{\mathrm{hor}}$. Let the obstacle have current estimated center $c_{o}\left(0\right)$, radius $r_{o}$, velocity estimate $v_{o}={\left(v_{x},v_{y}\right)}^{⊤}$, and velocity variances $\sigma_{v_{x}}^{2}$ and $\sigma_{v_{y}}^{2}$. Time is discretized using a fixed spatial step $\delta_{s}>0$:

$$\Delta t=\frac{\delta_{s}}{\max(∥v_{o}∥,\epsilon )},\;t_{k}=k\;\Delta t,\;k=0,\ldots ,N,\;t_{N}\leq T_{\mathrm{hor}}.$$

At each $t_{k}$, the mean position is propagated with a constant-velocity model,

$$c_{o}\left(t_{k}\right)=c_{o}\left(0\right)+v_{o}\;t_{k},$$

and the positional variances grow due to velocity uncertainty as

(4) $$\sigma_{o,x}^{2}\left(t_{k}\right)=\sigma_{o,x}^{2}\left(0\right)+t_{k}^{2}\;\sigma_{v_{x}}^{2},\;\sigma_{o,y}^{2}\left(t_{k}\right)=\sigma_{o,y}^{2}\left(0\right)+t_{k}^{2}\;\sigma_{v_{y}}^{2}.$$

To account for limited reaction capability, the effective collision radius is augmented with a time-decreasing safety margin:

(5) $$R\left(t_{k}\right)=r_{\mathrm{rob}}+r_{o}+\max\;\left(0,∥v_{o}∥\left(T_{\mathrm{react}}-t_{k}\right)\right).$$

Here $T_{\mathrm{react}}$ is a reaction-time parameter: at $t_{k}=0$, a full speed-dependent buffer is added, and this buffer decreases linearly until vanishing once $t_{k}$ exceeds $T_{\mathrm{react}}$.

Since the collision-probability map is computed before the robot’s future trajectory is known, every cell is treated as a potential robot position. However, not all obstacle predictions are physically reachable: if the robot’s maximum speed is $v_{\max}$, the farthest it can travel in $t_{k}$ seconds is $v_{\max}t_{k}$. Combined with the obstacle motion, the maximum closing distance is $d_{\mathrm{reach}}\left(t_{k}\right)=\left(v_{\max}+∥v_{o}∥\right)t_{k}$. When the predicted obstacle position lies beyond this frontier relative to the current robot position $\left(x_{r},y_{r}\right)$, the collision contribution is attenuated by an exponential soft gate:

$$w_{\mathrm{reach}}\left(t_{k}\right)=\left\{\begin{array}{cc} 1,\; & \mathrm{if}\;∥c_{o}\left(t_{k}\right)-{\left(x_{r},y_{r}\right)}^{⊤}∥\leq d_{\mathrm{reach}}\left(t_{k}\right),\\ \exp\;\left(-\left(∥c_{o}\left(t_{k}\right)-{\left(x_{r},y_{r}\right)}^{⊤}∥-d_{\mathrm{reach}}\left(t_{k}\right)\right)\right),\; & \mathrm{otherwise}. \end{array}\right.$$

A soft gate is preferred over a hard cut-off because the obstacle position is itself uncertain: the predicted center $c_{o}\left(t_{k}\right)$ may lie outside the reachability frontier, while the true position, drawn from the growing uncertainty ellipse of (4), may still fall within it. At each $t_{k}$, the per-cell collision probability is computed via (3), using the propagated obstacle state $\left(c_{o}\left(t_{k}\right),\sigma_{o,x}^{2}\left(t_{k}\right),\sigma_{o,y}^{2}\left(t_{k}\right)\right)$ and effective radius $R(t_{k})$ in place of $r_{\mathrm{rob}}+r_{o}$. The per-obstacle dynamic collision probability is the maximum weighted value over the prediction horizon:

$$P_{\mathrm{coll}}\left(c|o\right)=\max_{k}\left(P_{\mathrm{coll}}\left(c|o,t_{k}\right)\;w_{\mathrm{reach}}\left(t_{k}\right)\right),$$

and dynamic obstacles are aggregated via (1).

### 4.4. Extended-Footprint Correction

**Figure 2:**
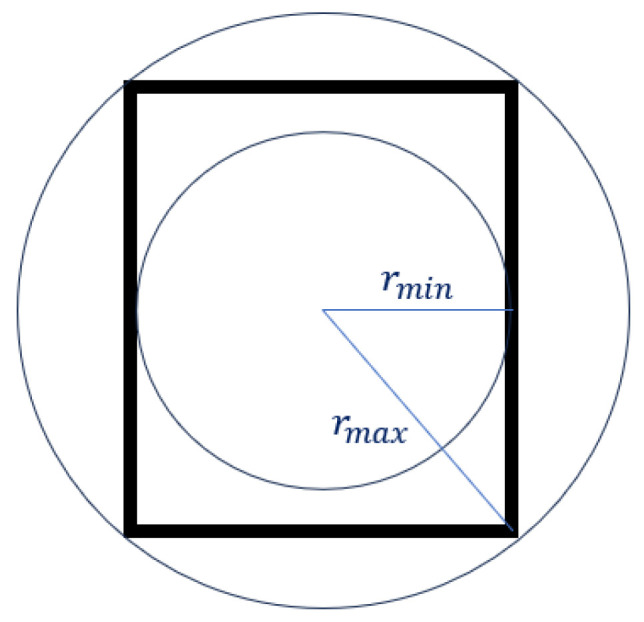
Minimum and maximum radius for a robot with a non-circular footprint.

The formulas above model the robot as a disc of radius $r_{\mathrm{rob}}$, which corresponds to the inscribed circle of the actual, generally non-circular, footprint. To account for the parts of the footprint that extend beyond this disc, we perform a second evaluation pass using an enlarged radius $r_{\max}>r_{\mathrm{rob}}$, corresponding to the circumscribed circle (Figure 2).

For each obstacle, the collision probability obtained with $r_{\max}$ is scaled by a factor $\alpha_{r}\in \left(0,1\right)$ to attenuate its contribution, since the robot does not occupy the full circumscribed disc. The nominal and extended results are combined as

$$P_{\mathrm{coll}}\left(c\right)\leftarrow \max\;\left(P_{\mathrm{coll}}^{r_{\mathrm{rob}}}\left(c\right),\min\;\left(P_{\mathrm{hi}},P_{\mathrm{coll}}^{r_{\max}}\left(c\right)\right)\right),$$

where $P_{\mathrm{coll}}^{r_{\max}}\left(c\right)$ is aggregated from the $\alpha_{r}$-scaled per-obstacle probabilities through the same union rule, and $P_{\mathrm{hi}}$ is a saturation threshold preventing the extended pass from dominating the risk estimate. This correction is applied independently to the static and dynamic aggregates, and the extended pass is only evaluated when the nominal probability lies below $P_{\mathrm{hi}}$, providing early termination for cells already at high risk.

### 4.5. Visibility and Occlusion Memory

To increase the robustness of the proposed approach and to account for the risk component associated with occlusion, we use a visibility mask to maintain local memory of previously detected collision risk, even when the area near a cell is no longer visible because it is hidden by other obstacles.

A cell $\left(x_{c},y_{c}\right)$ is marked as non-visible if the line of sight from the robot position $\left(x_{r},y_{r}\right)$ to the cell center intersects any detected obstacle. Cells within a minimum distance $d_{\min}$ from the robot are always considered visible. The line of sight is parameterized as

$$\gamma \left(t\right)=\left(1-t\right){\left(x_{r},y_{r}\right)}^{⊤}+t{\left(x_{c},y_{c}\right)}^{⊤},\;t\in \left[0,1\right].$$

For a segment obstacle with endpoints $\left(p^{f},p^{l}\right)$, occlusion occurs if there exist (t,u) such that

$$\gamma \left(t\right)=p^{f}+u\left(p^{l}-p^{f}\right),\;t\in \left(0,1\right),\;\;u\in \left[0,1\right].$$

For a circular obstacle with center $c_{o}$ and radius $r_{o}$, occlusion is detected by solving

$${∥\gamma \left(t\right)-c_{o}∥}^{2}=r_{o}^{2}$$

for t∈(0,1); the cell is occluded if a real solution exists in this interval.

The occlusion mask is constructed only from obstacles that are currently classified as static.

This distinction avoids creating shadows behind dynamic obstacles. If a moving obstacle temporarily passes between the robot and a region of the map, the cells behind it are not marked as occluded by the visibility module, and the monotonic memory update is not activated for those cells. The occlusion mask is recomputed at each risk-map update from the current obstacle estimates and robot pose. In our implementation, both the visibility check and the risk-map memory update run at 10Hz.

An occlusion buffer of radius $r_{\mathrm{occ}}$ is grown outward from occluded cells in the direction away from the robot using a directional breadth-first search, so that cells immediately behind an obstacle are treated conservatively even if they are not geometrically shadowed. For cells in the occluded or buffered region, the stored collision probability can only increase:

$$P_{\mathrm{coll}}\left(c\right)\leftarrow \max\;\left(P_{\mathrm{coll}}^{\mathrm{prev}}\left(c\right),P_{\mathrm{coll}}^{\mathrm{new}}\left(c\right)\right).$$

The monotonic update is applied only to the set of cells that are currently marked as non-visible by the visibility computation. Since this set is recomputed at every map update and moving obstacles are not used as occluders, the memory mechanism does not permanently preserve risk behind transient dynamic obstacles.

## 5. Risk Area Maps

In addition to collision risk, the system incorporates environment regions associated with elevated operational risk, such as areas with reduced localization reliability [52]. These risk areas are assumed known a priori and are represented as axis-aligned rectangles in the map frame, each defined by center $c_{a}={\left(X_{a},Y_{a}\right)}^{⊤}$ and dimensions $\left(W_{a},H_{a}\right)$. Because risk areas are defined in the map frame, in this case is essential to incorporate localization uncertainty in the violation probability estimation.

Let $\sigma_{\mathrm{loc},x}$ and $\sigma_{\mathrm{loc},y}$ be the standard deviations returned by the localization filter, for example, AMCL. We use the conservative isotropic proxy

$$\sigma_{\mathrm{loc}}=\max\left(\sigma_{\mathrm{loc},x},\;\sigma_{\mathrm{loc},y}\right).$$

The total positional uncertainty used for risk-area evaluation combines localization uncertainty and the uniform actuation-induced spread introduced in Section 3.2:

$$\sigma_{\mathrm{area}}=\sqrt{\sigma_{\mathrm{act},\mathrm{uni}}^{2}+\sigma_{\mathrm{loc}}^{2}},$$

where $\sigma_{\mathrm{act},\mathrm{uni}}$ denotes the fixed-horizon actuation uncertainty proxy used for global position evaluation in the map frame. We use this uniform quantity rather than the per-cell estimate of (2), because the risk-area probability depends on the robot global position in the map frame rather than on relative robot–obstacle geometry.

For a cell center $\left(x_{c},y_{c}\right)$ transformed into the map frame, the probability that the robot lies inside area *a* is approximated by axis-wise independence:

$$P_{\mathrm{inside}}\left(c,a\right)=P_{x}P_{y},$$

with

$$\begin{array}{cc} P_{x}\; & =\Phi \;\left(\frac{X_{a}+W_{a}/2-x_{c}}{\sigma_{\mathrm{area}}}\right)-\Phi \;\left(\frac{X_{a}-W_{a}/2-x_{c}}{\sigma_{\mathrm{area}}}\right),\\ P_{y}\; & =\Phi \;\left(\frac{Y_{a}+H_{a}/2-y_{c}}{\sigma_{\mathrm{area}}}\right)-\Phi \;\left(\frac{Y_{a}-H_{a}/2-y_{c}}{\sigma_{\mathrm{area}}}\right). \end{array}$$

When multiple areas of the same type $\left\{a_{1},\ldots ,a_{M}\right\}$ are present, the probability of being inside at least one (assuming independence) is

$$P_{\mathrm{area}}\left(c\right)=1-\prod_{j=1}^{M}\left(1-P_{\mathrm{inside}}\left(c,a_{j}\right)\right).$$

### Weighted Local Risk Map

The collision-probability map of Section 4 and the risk-area maps introduced in this section represent different sources of local navigation risk. Since the individual layers do not all share the same semantic meaning, the final map is interpreted as a risk-cost representation rather than as a globally calibrated probability field. Let M(c) denote the collision-related map value at cell *c*, and let $R_{i}\left(c\right)$ denote the *i*-th additional risk-area layer. Each layer is first scaled by a non-negative weight reflecting its relative severity. The final local risk map is then defined as

(6) $$R_{l}\left(c\right)=\max\left(w_{1}M\left(c\right),\;\max_{i}\;w_{i}R_{i}\left(c\right)\right).$$

where the non-negative weights $w_{1}$ and $w_{i}$ reflect the relative severity of different risk sources and can be tuned depending on the application. The max operator ensures that the most critical risk source dominates the local decision. This aggregation is not intended to yield a globally calibrated probability value, since collision risk, restricted-area violation risk, and task-level risk do not necessarily share the same semantics. The maximum is instead used as a conservative planning heuristic, ensuring that a high-risk contribution is not diluted by lower-risk layers.

## 6. Risk-Aware Planning

Once the local risk map has been constructed, it can be translated into motion decisions. As shown in the right-hand portion of Figure 1, we propose a local planning architecture in two steps. First, a local replanning module modifies a prefix of the current global path using the local risk map. Second, the resulting path is passed to the Model Predictive Path Integral (MPPI) controller, which, also using the risk map, remains responsible for path tracking. In this way, the controller operates on references that already reflect the local safety assessment.

The local replanning module receives as input the final weighted local risk map produced by the aggregation process described in the previous section. Let $\tilde{r}\left(c\right)\in \left[0,1\right]$ denote the normalized value of this map at cell *c*, where larger values correspond to higher local risk.

For graph search, we do not use $\tilde{r}\left(c\right)$ directly as the additive path cost. Instead, we apply the monotone transformation

(7) $$r\left(c\right)=\beta_{o}\log\;\left(\frac{1}{1-\min(\tilde{r}\left(c\right),\bar{r})}\right),$$

where $\beta_{o}>0$ is a scaling coefficient and $\bar{r}\in \left(0,1\right)$ is a saturation threshold.

This transformation preserves the ordering of the original map values, keeps the path cost additive, and penalizes high-risk cells more sharply.

### 6.1. Local A* Path Modification

Rather than replanning the full global route, the proposed method modifies only a local prefix of the reference path. At each replanning cycle, the active reference is chosen as either the most recent global plan or the previously accepted locally modified path, with periodic refresh from the global planner. The robot pose and the active reference waypoints are transformed from the map frame to the odometry frame, where the local risk map is defined, and only the suffix ahead of the robot is retained.

The replanning algorithm proceeds as follows: (i) a local goal is selected on the current reference path; (ii) the current local segment is checked against the risk map; (iii) if needed, a time-limited A*-based search is run in the odometry frame and (iv) the new segment is accepted only if it improves sufficiently over the current one according to the divergence-aware criterion defined below.

Let $\tau_{r}$ denote the admissibility threshold on the search cost, $d_{\mathrm{track}}$ the maximum allowed distance between the robot and the current reference path, and $N_{\mathrm{safe}}$ the maximum number of consecutive cycles for which a safe reference may be preserved without invoking a new local search. If all inspected waypoints satisfy $r\left(c\right)\leq \tau_{r}$ and the robot remains within $d_{\mathrm{track}}$ of the current path, the reference is preserved for at most $N_{\mathrm{safe}}$ consecutive cycles before replanning is imposed.

When a new global plan is received while the robot is farther than a bridging distance $d_{\mathrm{bridge}}$ from that plan, the straight bridge connecting the robot to the closest waypoint is explicitly checked; if this bridge is unsafe, local replanning is forced. When replanning is triggered, a local goal is selected along the reference path using a lookahead distance $d_{\mathrm{LA}}$. If the selected goal lies in a cell whose risk exceeds a threshold $\tau_{g}$, it is displaced to the nearest cell whose risk is below a safer threshold $\tau_{f}$.

#### 6.1.1. Additive Cost Formulation

Local replanning is performed on a grid graph, allowing both axial and diagonal transitions in the odometry frame. The search minimizes the additive objective

(8) $$J\left(\pi \right)=\sum_{k=0}^{N-1}\left[d\left(c_{k},c_{k+1}\right)+r\left(c_{k+1}\right)+\rho_{\mathrm{dir}}\left(c_{k},c_{k+1}\right)\right],$$

where $\pi =(c_{0},\ldots ,c_{N})$ denotes the candidate local path, d(·,·) is the Euclidean step length on the grid, and $\rho_{\mathrm{dir}}$ is a direction-dependent penalty.

Near the start node, cells not aligned with the current robot heading are penalized in order to stabilize the initial portion of the locally modified path. This reduces oscillatory replanning, for example, when an obstacle lies directly in front of the robot and successive replanning cycles would otherwise alternate between passing it on the left or on the right.

For a transition from node *i* to a neighboring node *j*, the incremental edge cost is

(9) $$g_{i\to j}=d_{i,j}+r\left(c_{j}\right)+\rho_{\mathrm{dir}}\left(i,j\right),$$

where $d_{i,j}=\delta \sqrt{\Delta x^{2}+\Delta y^{2}}$ is the Euclidean step length and δ is the map resolution. The heuristic term is the Euclidean distance to the local goal,

(10) $$h(j)=d\;\left(j,\;c_{\mathrm{goal}}\right),$$

so node expansion is driven by the standard score

(11) f(j)=g(j)+h(j).

The local search is run for at most $T_{\max}$ seconds. If the local goal is not reached within this budget, the path is reconstructed toward the explored node with the smallest heuristic value, which acts as a surrogate local goal.

#### 6.1.2. Path Acceptance Criterion

Let $C_{\mathrm{old}}$ and $C_{\mathrm{new}}$ denote the additive costs of the current local segment and the replanned segment, respectively, both evaluated in the odometry frame. The current segment is defined as the prefix of the active reference path extending from the robot to the selected lookahead point, whereas the candidate segment is the output of the local search. Their spatial divergence $\delta_{\mathrm{div}}$ is computed as the ξ-quantile of point-to-nearest-point distances, where ξ∈(0,1] is a design parameter.

The new segment is accepted only if

(12) $$C_{\mathrm{new}}\leq \left(1-\eta (\delta_{\mathrm{div}})\right)\;C_{\mathrm{old}},$$

where

(13) $$\eta \left(\delta_{\mathrm{div}}\right)=\left\{\begin{array}{cc} \eta_{\min}, & \delta_{\mathrm{div}}\leq d_{\min},\\ \eta_{\max}, & \delta_{\mathrm{div}}\geq d_{\max},\\ \eta_{\min}+{\left(\frac{\delta_{\mathrm{div}}-d_{\min}}{d_{\max}-d_{\min}}\right)}^{\gamma_{\eta }}\left(\eta_{\max}-\eta_{\min}\right), & \mathrm{otherwise}. \end{array}\right.$$

Here, $\eta_{\min}$ and $\eta_{\max}$ are the minimum and maximum improvement ratios, $d_{\min}$ and $d_{\max}$ are divergence breakpoints, and $\gamma_{\eta }$ shapes the interpolation between them.

In addition, the candidate segment is force-accepted whenever the robot is farther than $d_{\mathrm{acc}}$ from the path it is currently following. When a new segment is accepted, it is transformed back to the map frame, stitched to the remainder of the original global path after the closest waypoint to its last local point, and a tangent-based orientation profile is recomputed over the stitched path.

To avoid unnecessary controller perturbations, the updated path is republished only when publication is forced, when its divergence from the last published path exceeds a threshold $d_{\mathrm{pub}}$, or when a heartbeat interval $T_{\mathrm{hb}}$ expires.

### 6.2. Model Predictive Path Integral Control

The path tracking in the proposed architecture is handled by a Model Predictive Path Integral (MPPI) controller. MPPI is a sampling-based predictive controller that evaluates candidate control sequences over a finite horizon using a local map and a reference path. In this work, we use the MPPI implementation available in the ROS 2 Nav2 framework.

The main benefit of this integration is that the proposed risk-aware framework remains fully compatible with a standard navigation pipeline. The controller itself is left unchanged: MPPI receives the locally modified reference path produced by the replanning module, and the proposed risk map replaces the standard obstacle-inflation costmap. Therefore, risk awareness is introduced without modifying the internal MPPI objective or redesigning the downstream control architecture. This preserves the modular structure of standard costmap-based navigation stacks while enabling risk-aware local motion generation.

## 7. Validations

We validate the proposed framework through extensive simulations in Gazebo. To assess robustness with respect to uncertainty, we evaluate system performance under two levels of sensing and actuation degradation, referred to as *low uncertainty* and *high uncertainty* throughout this section. All simulations are conducted using a Robotnik Summit XL-Steel mobile robot equipped with two SICK microScan3 LiDAR sensors, mounted at the front and rear of the platform (Robotnik Summit XL-Steel simulator: https://github.com/RobotnikAutomation/summit_xl_sim (accessed on 15 June 2026)). The robot has a rectangular footprint of 0.78m×0.98m.

### 7.1. Simulation Setup

We use a hybrid Robot Operating System (ROS) architecture. Perception, tracking, risk estimation, and planning modules run in ROS 1, while the MPPI controller is taken from the ROS 2 Navigation stack (Nav2). Communication between the two systems is handled through a ROS 1–ROS 2 bridge, which transfers costmaps, sensor measurements, and control commands.

Sensor uncertainty is introduced by modifying the LiDAR sensor model in the robot URDF, increasing the standard deviation of range measurements to simulate degraded sensing conditions. Actuation disturbances are modeled as an additive perturbation applied to the nominal velocity command before it reaches the low-level controller. For each velocity component i∈{v,ω}, the corrupted command is

$$u_{i}=u_{i}^{\mathrm{nom}}+{\tilde{w}}_{i}+b_{i},$$

where ${\tilde{w}}_{i}=\max\left(-{\bar{n}}_{i},\;\min\left(w_{i},\;{\bar{n}}_{i}\right)\right)$ is a zero-mean Gaussian sample $w_{i}∼N\left(0,\sigma_{i}^{2}\right)$ truncated to the symmetric interval $\left[-{\bar{n}}_{i},\;{\bar{n}}_{i}\right]$, accounting for high-frequency stochastic disturbances. The bias $b_{i}$ is driven by an Ornstein–Uhlenbeck (OU) process, which introduces temporally correlated drift meant to capture persistent effects such as unmodeled friction or drive asymmetries. Its exact discretization is

$$b_{i}\left(t+\Delta t\right)=\alpha_{i}\;b_{i}\left(t\right)+\sigma_{b,i}\sqrt{1-\alpha_{i}^{2}}\;\xi ,\;\alpha_{i}=e^{-\Delta t/\tau_{i}},\;\xi ∼N\left(0,1\right),$$

so that $b_{i}$ converges to a stationary distribution $N(0,\sigma_{b,i}^{2})$ with correlation time $\tau_{i}$, regardless of the control rate. When the commanded velocity is near zero, the bias state is reset and noise injection is suspended, preventing the robot from drifting while nominally at rest. Table 3 reports the parameters used in the two uncertainty conditions.

For comparison, we evaluate the proposed approach against a baseline navigation stack composed of a standard ROS 1 global planner (move_base) combined with the ROS 2 MPPI local planner operating on conventional costmaps. In the baseline configuration, the local costmap includes the standard obstacle and inflation layers, as well as a custom layer identifying risk areas to be avoided. To evaluate the effect of different safety margins around obstacles, we run the baseline with multiple inflation radii (0.55, 0.65, and 0.85m). Furthermore, to isolate the contribution of the uncertainty-aware local maps, we also evaluate in the cluttered scenario the standard Nav2 costmap configuration with only the local path modifier enabled.

The global planner recomputes the path from the current robot position at 0.1 Hz. The A* local path modifier, when enabled, runs at 2 Hz but publishes a new path only when a significantly better one is found, as described in Section 6.1. The MPPI controller runs at 10 Hz.

**Figure 3:**
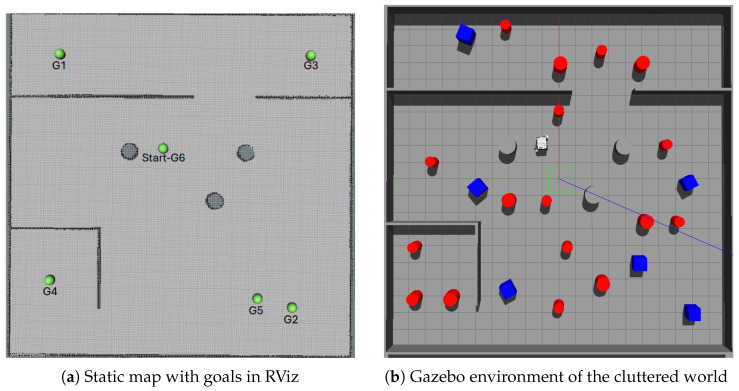
Representation of the base world map and the cluttered Gazebo world. (**a**) Static occupancy map visualized in RViz, with the green markers indicating the navigation goals. (**b**) The Gazebo world of the cluttered scenario. The colored red, green, and blue axes indicate the displayed reference frame, while the red cylinders and blue parallelepipeds represent the additional static obstacles present in the simulated environment but not included in the offline map.

The approach is tested in three environmental scenarios with increasing complexity, all sharing the same static map shown in Figure 3a. In each scenario, several static and/or dynamic obstacles that were not present during the offline mapping phase are introduced. In all experiments, the robot starts from the location marked “start” in the map and navigates through a fixed goal sequence $G_{1}\to G_{2}\to G_{3}\to G_{4}\to G_{5}\to G_{6}$. The sequence is repeated for ten iterations to collect statistically meaningful data. These experiments are designed to evaluate the robustness of the proposed approach in cluttered environments containing previously unseen obstacles. Accordingly, the global map is never updated during execution, even when obstacles can be classified as static. This choice reproduces the scenario of a robot entering an environment that changes frequently, where runtime map updates may be undesirable because the stored map could quickly become outdated. Repeating the goal sequence is intended solely for statistical evaluation, not to simulate a repetitive operational task.

In addition to collision-related risk, the environment contains two static risk areas, each assigned weight $w_{i}=0.8$ These areas are included in all scenarios and are used to evaluate whether the planner can balance collision avoidance against soft task-level constraints.

All experiments are executed on an Intel Core i7-1280P (12th Gen, 1.8 GHz base/4.8 GHz turbo) processor with 16 GB of RAM. The complete set of parameters used for simulations is available in the Supplementary Materials.

Collisions are detected using the Gazebo contact-sensor plugin attached to the robot footprint. This choice makes the collision metric independent of the internal collision-risk estimate used by the proposed framework and of the costmap representation used by the baselines. Restricted-area violations are evaluated using the Gazebo ground-truth pose of the robot with respect to the predefined restricted regions. Therefore, this metric is also independent of the risk-area evaluation module and reflects the actual simulated robot position.

A goal is counted as aborted when the navigation stack reports failure, for example, because the controller cannot find a feasible command or the recovery behaviors cannot restore a valid navigation state. After each collision or aborted goal, the robot is automatically reset to a predefined recovery pose before attempting the next goal in the sequence. This reset procedure ensures that all methods are evaluated under comparable conditions and that a failure in one attempt does not bias the initial condition of the subsequent attempt.

For each scenario, the same protocol is applied to the proposed risk-aware framework and to the standard MPPI baselines with fixed inflation radii. The reported collision counts, abort counts, success rates, completion times, and restricted-area violations are computed over the resulting set of navigation attempts.

To assess whether the differences in results between the proposed framework and the baselines are statistically significant, we apply a two-sided Fisher’s exact test to the per-run binary outcomes. For each metric, every navigation attempt is coded as 1 if at least one event of that type (collision, abort, or restricted-area violation) occurs during the run, and 0 otherwise. The binary outcomes are pooled across all goals of a scenario, and the proposed framework is compared against each baseline configuration; comparisons among baselines are not considered. Differences are reported as significant when p<0.05.

### 7.2. Cluttered Environment

**Figure 4:**
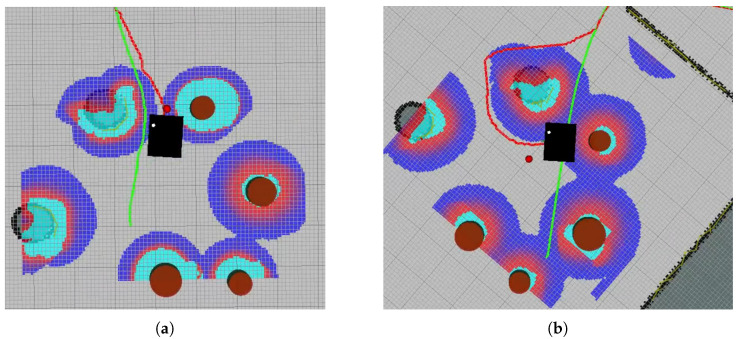
Local risk map in the cluttered scenario under different uncertainty levels. The black rectangle indicates the robot footprint. The green line is the global plan; the red line is the locally modified path. Risk values are visualized using the standard RViz costmap color scale, where blue indicates low risk, red indicates high risk, and cyan indicates near collision values. (**a**) Low uncertainty conditions. (**b**) High uncertainty conditions.

The first scenario is a highly cluttered but static environment, shown in Figure 3b. The three grey cylinders are known static obstacles already present in the global map (Figure 3a). The red cylinders and the blue parallelepipeds are unknown obstacles that the detection module detects and tracks as circles. Figure 4a,b illustrate the local risk map under low and high uncertainty conditions, respectively. The known cylinders are classified as segments since they belong to the static map. In Figure 4a, the obstacle on the middle-right is assigned a higher uncertainty than the surrounding ones despite the overall low-uncertainty setting, because it is a parallelepiped and so not perfectly approximated by the cylindrical model adopted by the detector, which can lead to lower measurement coherence across observations.

Table 4 compares the full risk-aware framework against the standard MPPI controller operating on conventional costmaps without local path modification. For each goal, the table reports the number of collisions (*coll.*), the number of aborted goal executions (*aborts*), the success rate (*succ.*), and the time required to reach the goal (*time* [s]). Here, an *abort* denotes a goal execution in which the controller fails at least once to compute a feasible solution because the robot is too close to obstacles, even after the recovery behaviors provided by Nav2. Multiple abort events during the same goal execution are counted only once in the table. If more than five aborts occur while pursuing the same goal, the robot is considered stuck, that goal is marked as failed, and execution proceeds to the next goal, analogously to the collision case. The reported time is computed only over successful goal executions.

The results show that the environment is too cluttered for the MPPI controller alone: regardless of the inflation radius, the controller remains closely tied to the global plan, which is computed on the outdated static map and, therefore, passes through narrow or obstructed passages. This leads to frequent controller infeasibility events and, in several cases, to failed goals. In contrast, the risk-aware framework achieves a 100% success rate on all goals with zero collisions.

The reduction in aborted executions achieved by the proposed framework is statistically significant with respect to both standard MPPI baselines (two-sided Fisher’s exact test, p<0.05).

#### Effect of the Local Risk Map

Since the standard MPPI alone cannot handle this level of clutter (Table 4), we isolate the contribution of the proposed risk map by integrating the local path modifier into the standard pipeline while retaining conventional costmaps. This configuration uses the same MPPI costmap for planning but adds the A*-based path modification described in Section 6.1. We test this configuration with three inflation radii (0.55, 0.65, and 0.85 m).

Table 5 and Table 6 show that the 0.55 m inflation radius produces both collisions and aborts, confirming that a small fixed margin is insufficient in this cluttered layout. The 0.65 m radius eliminates collisions and achieves a high success rate with shorter completion times, representing the best-performing standard baseline. However, the 0.85 m radius performs worse than 0.65 m despite its larger margin. This counter-intuitive result is caused by the absence of occlusion memory in the standard costmap: as obstacles enter and leave the sensor field of view, the safest path changes continuously, causing oscillatory replanning that degrades overall performance. Using a maximum-based costmap update rule would preserve memory of past detections, but it would also retain occupied cells for dynamic obstacles long after they have moved away, eventually making planning infeasible. The proposed risk map avoids this issue through its visibility-based memory mechanism (Section 4.5), which selectively preserves risk only in occluded regions.

In contrast, the risk-aware framework (left columns of Table 5) achieves zero collisions and near-zero aborts on all goals under low uncertainty, with completion times comparable to the best standard baseline (0.65 m).

The reduction in aborted executions achieved by the proposed framework is statistically significant with respect to the replanning baseline with the 0.55 and 0.85 m inflation radius, while the comparison against the 0.65 m replanning baseline is not significant.

**Figure 5:**
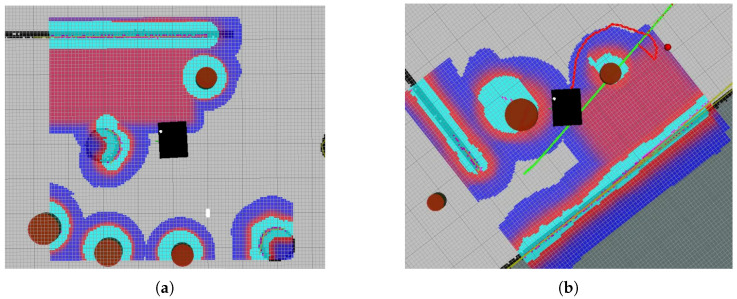
Local risk map in proximity of the two static risk areas. The black rectangle indicates the robot footprint. Risk values are visualized using the standard RViz costmap color scale, where blue indicates low risk, red indicates high risk, and cyan indicates near-collision values. (**a**) Risk area encountered along the path from the start to Goal 1. (**b**) Risk area encountered during navigation toward Goals 4 and 5.

Table 5 and Table 6 also report the number of risk-area violations. These results highlight the trade-off between collision avoidance and soft task-level risk. The standard planners with small inflation radius tend to cut through the risk area encountered on the way to Goal 1, whereas the proposed risk-aware planner occasionally enters the lower risk area encountered during navigation toward Goals 4 and 5. In that part of the environment, a narrow passage is formed between the obstacle and the risk area (Figure 5b); because collision-related risk is weighted more strongly than the static risk-area penalty, the planner prefers to keep a safer distance from the obstacle, even at the cost of moving closer to the risk area.

The standard planner with inflation radius 0.85 m sometimes enters both risk areas. Near Goal 1, this happens because the standard approach does not inflate the risk-area layer itself; near Goals 4 and 5, the larger inflation radius pushes the robot farther from the obstacle, and therefore, closer to the nearby risk area.

Table 7 reports the results under high uncertainty conditions, comparing the risk-aware framework against the best-performing standard baseline (0.65 m inflation with local path modification). Although both configurations produce zero collisions, the standard pipeline accumulates 14 aborts across all goals and fails to reach Goal $G_{4}$ in one run (success rate 0.90), whereas the risk-aware framework completes all goals without a single abort.

The risk-area statistics show a similar trend. Under increased uncertainty, the standard planner violates the Goal 1 risk area more frequently, whereas the proposed framework maintains a comparable number of violations in the lower risk area near Goals 4 and 5. This suggests that the proposed risk representation is more robust to uncertainty variations, adapting its collision-related caution without requiring manual retuning of fixed margins.

The reduction in aborts of the proposed framework relative to the 0.65 m replanning baseline is statistically significant (Fisher’s exact test, p<0.05).

**Figure 6:**
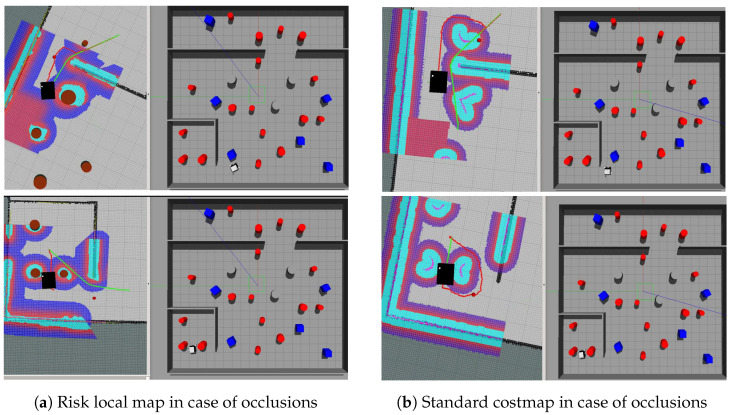
Visibility mask contribution. The black rectangle indicates the robot footprint, while the green and red lines denote the global plan and the locally modified path, respectively. The burgundy circles in the RViz panels indicate the circular obstacles identified by the detection module. The risk map is visualized using the standard RViz costmap color scale. (**a**) The local risk costmap keeps memory of risk values even in case of occlusion. (**b**) The standard costmap does not track occlusions. On the upper figures, the static risk area is visible.

The visibility mask enables a local memory of detected obstacles, preserving risk values even when cells are no longer visible due to occlusions from other obstacles. This mechanism is especially important in cluttered environments where substantial path modifications are required. Without such memory, as in the standard costmap, the planner may direct the robot toward a previously occupied area that has temporarily become invisible, as illustrated in Figure 6b. Figure 6a shows how the proposed risk map retains the collision probability behind occluding obstacles, preventing this unsafe behavior.

### 7.3. Moving Obstacles Environment

**Figure 7:**
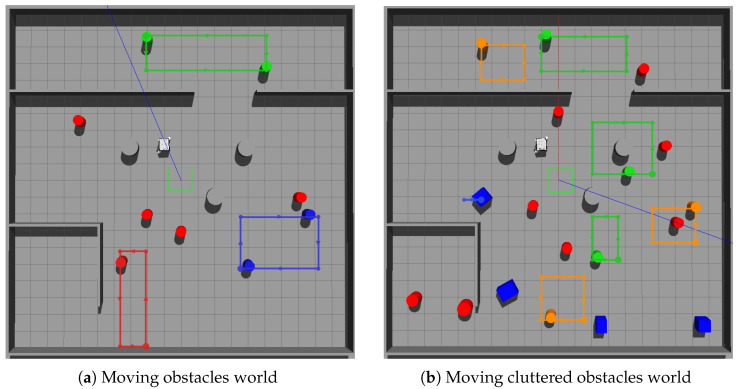
Gazebo worlds with moving obstacles. In (**a**), the cylindrical obstacles move along the rectangular trajectories shown in the scene. In (**b**), the green cylinders move continuously along the green trajectories, whereas the orange cylinders and the blue cuboid on the center-left perform periodic stop-and-go motions. The remaining red cylinders and blue cuboids represent additional static obstacles in the simulated environment.

The moving obstacles scenario contains five dynamic cylindrical obstacles that follow closed rectangular waypoint loops at speeds between 0.4 and 0.6 m/s. The dynamic obstacles are arranged in counter-circulating pairs (two obstacles sharing the same rectangular path but traveling in opposite directions) so that head-on and crossing encounters occur regularly along the corridors (Figure 7a). Because the waypoint controller steers each obstacle directly toward the next corner, direction changes are abrupt: the velocity vector rotates by 90° with no deceleration phase, producing instantaneous jumps in the heading. This scenario is designed to stress-test the detection and tracking pipeline under conditions of sudden motion discontinuities, frequent mutual occlusions between obstacles, and confined spaces where the robot has limited room to maneuver.

**Figure 8:**
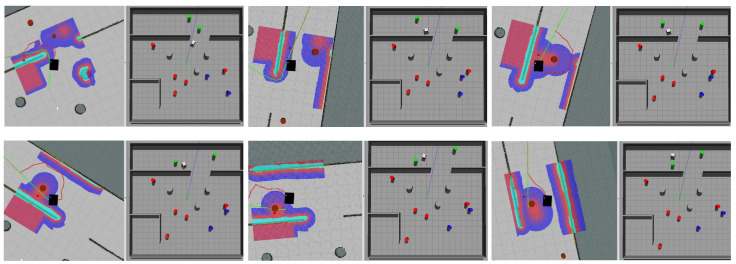
Evasive maneuver by the risk-aware framework: an obstacle abruptly changes direction toward the robot, which is constrained by a wall on the opposite side. The robot reverses and performs an evasive maneuver, successfully avoiding the collision. The black rectangle indicates the robot footprint, while the green and red lines denote the global plan and the locally modified path, respectively. The burgundy circles in the RViz panels indicate the circular obstacles identified by the detection module. The risk map is visualized using the standard RViz costmap color scale.

Table 8 and Table 9 report the results under low uncertainty conditions. The risk-aware framework (Table 8) achieves an average success rate above 0.90 across all goals, with a total of 5 collisions over 60 goal attempts. The remaining collisions occur when the detection module is not able to detect, identify, and correctly classify the obstacle in time or when it changes direction at the last moment in a constrained space. Figure 8 illustrates one such event in which the robot successfully performs a backward evasive maneuver despite being constrained by a wall.

In contrast, the standard MPPI baselines (Table 9) suffer dramatically: the 0.65 m inflation radius accumulates 28 collisions and 16 aborts, with goal $G_{4}$ reaching only 10% success rate. The 0.85 m radius reduces collisions to 22 but still produces 11 aborts, and goal $G_{3}$ drops to a 20% success rate. The standard controller lacks predictive obstacle propagation, making it unable to react early enough to fast-moving obstacles.

### 7.4. Cluttered and Dynamic Environment

The third scenario combines clutter with dynamic obstacles to create the most demanding test (Figure 7b). The environment contains fourteen static obstacles of mixed geometry: the pillars already present in the static map, red cylinders (radii 0.3–0.35 m), and rectangular boxes (sides 0.7–1.0 m). Six dynamic cylindrical obstacles (radius 0.3 m) are divided into two classes: three always-moving obstacles that continuously patrol rectangular loops at speeds of 0.4–0.5 m/s with abrupt 90° direction changes at waypoint corners, and three stop-and-go obstacles that remain stationary for 8–15 s before traversing a rectangular patrol at 0.3–0.4 m/s and stopping again. An additional stop-and-go box (0.8 m ×0.8 m) performs a short 1 m back-and-forth displacement every 15 s. The stop-and-go behavior is specifically designed to stress the dynamic/static classification: an obstacle that has been confidently classified as static will suddenly begin moving, and one that was tracked as dynamic will abruptly stop, requiring the hysteresis mechanism and the Mahalanobis-based criterion described in Section 3.1 to respond without excessive delay or false reclassifications.

Table 10 reports the results under high uncertainty conditions, comparing the risk-aware framework against the standard MPPI with a 0.65 m inflation radius.

The risk-aware framework accumulates 7 collisions over 60 goal attempts (average success rate 0.88), with zero aborts. The great majority of collisions occur when a previously stationary obstacle suddenly starts moving toward the robot, or when dynamic obstacles are erroneously classified as static. The standard baseline performs substantially worse: 34 collisions and 1 abort over 60 attempts, with goal $G_{2}$ reaching only a 10% success rate and goal $G_{1}$ at 20%. The reduction in collisions with respect to the standard MPPI baseline is statistically significant (Fisher’s exact test, p<0.05). This confirms that the proposed framework’s ability to differentiate uncertainty sources and adapt margins online provides a significant advantage in environments that combine heterogeneous obstacle types and motion patterns.

### 7.5. Computational Analysis

We profile the proposed framework during the simulation experiments using an external sampler that records the CPU usage and resident memory of every node at 2 Hz, together with the publication rate of the main output topic of each module. We report results for three scenarios of increasing environmental complexity: base (empty environment), cluttered (only static unknown obstacles), and cluttered with dynamic obstacles, all with the high-uncertainty configuration of Table 3. Each scenario is profiled over a full goal sequence, after dropping a 10 s warm-up window. CPU values are expressed as a percentage of one logical core; on the 20-core Intel Core i7-1280P used in the experiments, a value of 100% corresponds to 5% of the total compute budget.

Across all scenarios, the framework meets its design rates: the obstacle detection and tracking module runs at 12.50 Hz, the local risk map and the online uncertainty estimates are published at 9.97–10.00 Hz, and the MPPI velocity command is published at 9.28–9.64 Hz. The slightly lower wall-clock rate of the MPPI output reflects the Gazebo real-time factor on the simulation host, since the ROS 2 controller uses simulated time whereas the ROS 1 measurement is in wall clock.

Table 11 reports the per-module CPU and memory footprint of the proposed framework in the most demanding scenario (cluttered with dynamic obstacles, high uncertainty). The full pipeline requires on average 131% of one logical core, with the dominant contributions from the local A* path adaptation and from the risk-map construction stage. The infrastructure components shared with the baseline configuration (Gazebo, the MPPI controller, the behavior tree, the global planner, AMCL, and the ROS 1/ROS 2 bridge) are not included in the framework total.

Table 12 reports the framework CPU usage across the three scenarios. The total load increases monotonically with environmental complexity, from 107% to 131% of one logical core. The increase is concentrated in two modules whose work scales with the number of tracked obstacles: the risk-map construction stage (28.5%→37.6%, +32%) and the local A* path adaptation (33.5%→44.6%, +33%). The remaining modules (detection and tracking, laser merging, risk-area evaluation, and map fusion) remain essentially flat across scenarios. Memory usage is stable across scenarios, with per-module RSS ranging from 11 MB (map fusion) to 82 MB (local A* path adaptation), and a total framework footprint of 207 MB.

## 8. Limitations

The proposed framework should be interpreted as a practical, planning-oriented risk-cost representation rather than as a formally exact probabilistic model of collision risk. Several approximations are introduced to obtain a representation that can be computed online and used within standard costmap-based navigation pipelines. In particular, actuation uncertainty is represented through an isotropic spatial proxy, and heterogeneous risk layers are combined through a maximum operator. These choices are conservative and useful for local planning, but they do not provide formal approximation-error bounds or safety guarantees.

The current perception pipeline is based only on 2D LiDAR detections. This choice keeps the system lightweight and compatible with common mobile-robot sensing setups, but it limits the ability to detect obstacles outside the LiDAR scanning plane and does not provide semantic classification. In the dynamic experiments, the remaining collision cases are mainly caused by obstacles that are not classified as dynamic early enough, so their predicted motion is not incorporated into the risk map with sufficient anticipation. The framework is modular with respect to the perception front-end: visual detections, RGB-D information, or 3D LiDAR measurements could be integrated by providing additional obstacle detections, semantic labels, and associated uncertainty estimates to the same risk-map construction process. Such multi-sensor fusion could improve early moving-agent classification and increase robustness in partially observable scenes. Implementing and validating this extension is left for future work.

Dynamic obstacles are propagated using a constant-velocity model. This model is suitable as a lightweight baseline for real-time local risk-map construction, but it cannot capture abrupt maneuvers or multimodal human motion. More expressive predictors, including multimodal or learning-based human-motion models, could in principle replace the current propagation step by providing multiple predicted obstacle states with associated probabilities or uncertainty estimates. However, their benefit in the proposed costmap-based framework is not automatic: projecting several motion hypotheses into a local risk map may enlarge risk regions and reduce the free space available for evasive maneuvers. Therefore, advanced prediction models should be coupled with appropriate risk aggregation, prediction-horizon selection, and controller-level reactivity and require a dedicated evaluation.

The parameters of the proposed risk-map construction and local path adaptation are manually selected and kept fixed during the experiments to preserve interpretability and reproducibility. Although perception and actuation uncertainty are estimated online, other parameters, such as risk weights, saturation thresholds, smoothing coefficients, and path-acceptance thresholds, may require retuning across platforms and environments. Adaptive or learning-based parameter selection is, therefore, an important direction for future work. We did not perform a systematic sensitivity analysis over these parameters. Such an analysis would require a dedicated experimental campaign, since the effect of each parameter depends on the environment geometry, obstacle density, uncertainty level, dynamic-obstacle behavior, and controller response. Providing a sensitivity study and deriving general tuning guidelines are, therefore, left as future work.

Finally, the validation is conducted in Gazebo simulation. This allows controlled and repeatable evaluation under different sensing and actuation uncertainty conditions, but it does not eliminate the sim-to-real gap. Real-robot experiments, hardware-in-the-loop validation, and tests with richer perception pipelines are required before drawing conclusions about deployment in safety-critical real-world environments.

## 9. Conclusions and Future Works

This work has presented a framework that explicitly acknowledges and quantifies uncertainty at the reactive motion planning level. Unlike methods that provide formal guarantees, which can be overly conservative, the proposed approach enables a straightforward integration with state-of-the-art controllers such as MPPI. The perception module estimates detection uncertainty by analyzing the temporal coherence of measurements and evaluating the instantaneous explainability of the observations. This uncertainty is then incorporated into the local costmap, allowing the control module to adapt its behavior to the current level of uncertainty and to act more conservatively when required. Additionally, the planner modification enables the robot to navigate cluttered and dynamic environments without requiring updates to the global costmap. In the validation section, we demonstrated the effectiveness of each component of the framework in both static and dynamic scenarios.

Currently, dynamic obstacles are treated as generic obstacles, without explicitly considering the safety specifications required when the obstacle is a person. However, the proposed approach could be integrated with the ISO TS-15066 [53] Speed and Separation Monitoring (SSM) framework. Since the system already computes the distance between obstacles and the cells of the local map together with the associated uncertainty, the maximum allowable robot velocity in each cell could be determined according to the SSM formulation. At the moment, perception relies entirely on LiDAR measurements. This choice ensures a computationally efficient implementation, but performance could be improved by integrating camera-based obstacle detection. Future work will focus on estimating and using the uncertainty arising from the fusion of image and LiDAR information within the proposed framework. 

## Figures and Tables

**Table 1:** Qualitative comparison of the proposed framework with representative related approaches. ✓ = explicitly addressed, (✓) = partially addressed, i.e., considered only under simplifying assumptions, in a restricted setting, or indirectly rather than as a central and general mechanism, – = not addressed.

Method	Perception Uncert.	Online Uncert. Estim.	Actuation Uncert.	Dynamic Obstacle Pred.	Occlusion Reasoning	Heterog./Task-Level Risk	Probabilistic Costmap	Std. Planner Compat.
Blackmore et al. [4]	–	–	✓	–	–	–	–	–
Fulgenzi et al. [20]	✓	✓	–	✓	(✓)	–	(✓)	–
Patil et al. [26]	✓	(✓)	✓	–	–	–	–	(✓)
Hakobyan et al. [6]	–	(✓)	–	✓	–	–	–	–
Laconte et al. [28]	✓	✓	–	✓	–	✓	✓	–
Nishimura et al. [10]	–	✓	–	✓	–	–	–	–
Firoozi et al. [9]	(✓)	(✓)	–	✓	✓	–	–	–
Weber et al. [43]	–	–	–	–	–	✓	–	✓
Trevisan et al. [22]	(✓)	(✓)	–	✓	–	–	–	–
Proposed	✓	✓	✓	✓	✓	✓	✓	✓

**Table 2:** Navigation risk factors, causes, and corresponding framework components.

Risk Factor	Cause	Framework Component
Collision	Sensor occlusion Obstacle motion uncertainty Sensing noise Actuation uncertainty	Detection Collision probability map Risk-aware planning
Restricted areas violation	Localization uncertainty Actuation uncertainty	Risk area maps Risk-aware planning
Task aborted	Robot too close to obstacles Robot stuck	Collision probability map Risk-aware planning

**Table 3:** Noise parameters for the low- and high-uncertainty scenarios.

Source	Parameter	Low	High
LiDAR	$\sigma_{\mathrm{range}}$ [m]	0.02	0.06
Actuation	$\sigma_{v}$ [m/s]	0.003	0.05
	$\sigma_{\omega }$ [rad/s]	0.003	0.05
	$\sigma_{b,v}$ [m/s]	0.015	0.09
	$\sigma_{b,\omega }$ [rad/s]	0.015	0.08
	$\tau_{v},\;\tau_{\omega }$ [s]	2.0	5.0
	${\bar{n}}_{v}$ [m/s], ${\bar{n}}_{\omega }$ [rad/s]	0.05, 0.05	0.5, 0.4

**Table 4:** Cluttered scenario under low uncertainty. Comparison of the proposed risk-aware framework against the standard MPPI controller (without local path modification) using conventional costmaps with two inflation radii. For each goal, *coll.* denotes the number of collisions, *aborts* the number of goal executions in which the controller became infeasible at least once, *succ.* the fraction of successful executions, and *time* the time to reach the goal, reported as mean ± standard deviation over successful runs only.

	Risk-Aware Framework				Standard MPPI, Infl. 0.65 m				Standard MPPI, Infl. 0.85 m			
Goal	Coll.	Aborts	Succ.	Time [s]	Coll.	Aborts	Succ.	Time [s]	Coll.	Aborts	Succ.	Time [s]
1	0	0	1.00	25.90 ± 0.21	0	5	1.00	23.56 ± 0.90	0	8	0.70	24.63 ± 0.97
2	0	0	1.00	42.43 ± 0.89	0	8	1.00	34.96 ± 1.01	0	8	0.90	34.19 ± 1.75
3	0	0	1.00	38.23 ± 1.32	0	7	1.00	32.30 ± 0.81	0	8	1.00	32.67 ± 0.93
4	0	0	1.00	51.80 ± 1.25	0	10	0.70	48.30 ± 1.90	0	10	0.70	47.05 ± 3.68
5	0	0	1.00	28.33 ± 1.23	0	9	1.00	33.40 ± 2.69	0	10	0.90	30.13 ± 1.02
6	0	1	1.00	19.46 ± 0.74	0	1	1.00	17.21 ± 0.81	0	0	1.00	16.72 ± 0.55

**Table 5:** Cluttered scenario, low uncertainty. Risk-aware framework versus standard costmap with local path modification and inflation radius 0.55 m.

	Risk-Aware Framework					Standard with Replanning, Infl. 0.55 m				
Goal	Collisions	Aborts	Area Violations	Success Rate	Time	Collisions	Aborts	Area Violations	Success Rate	Time
1	0	0	1	1.00	25.90 ± 0.21	3	0	4	0.70	25.45 ± 0.47
2	0	0	0	1.00	42.43 ± 0.89	0	3	0	1.00	40.97 ± 1.29
3	0	0	0	1.00	38.23 ± 1.32	1	2	0	0.80	34.52 ± 1.38
4	0	0	4	1.00	51.80 ± 1.25	2	0	0	0.80	48.67 ± 1.91
5	0	0	2	1.00	28.33 ± 1.23	5	2	0	0.50	34.99 ± 1.65
6	0	1	0	1.00	19.46 ± 0.74	0	2	0	1.00	18.42 ± 0.56

**Table 6:** Cluttered scenario, low uncertainty. Standard costmaps with local path modification and inflation radii 0.65 m and 0.85 m.

	Standard with Replanning, Infl. 0.65 m					Standard with Replanning, Infl. 0.85 m				
Goal	Collisions	Aborts	Area Violations	Success Rate	Time	Collisions	Aborts	Area Violations	Success Rate	Time
1	0	0	3	1.00	24.65 ± 0.31	0	0	7	1.00	25.47 ± 0.27
2	0	1	0	1.00	40.07 ± 0.50	0	0	0	1.00	50.83 ± 4.92
3	0	0	0	1.00	31.68 ± 0.39	0	1	0	1.00	44.29 ± 1.60
4	0	0	0	1.00	46.15 ± 0.40	0	4	5	1.00	70.25 ± 4.63
5	0	0	1	1.00	27.19 ± 0.95	0	1	6	1.00	29.17 ± 0.96
6	0	0	0	1.00	17.48 ± 0.11	0	3	0	0.70	46.64 ± 10.23

**Table 7:** Cluttered scenario, high uncertainty. Risk-aware framework versus standard costmap with local path modification and inflation radius 0.65 m.

	Risk-Aware Framework					Standard with Replanning, Infl. 0.65 m				
Goal	Collisions	Aborts	Area Violations	Success Rate	Time	Collisions	Aborts	Area Violations	Success Rate	Time
1	0	0	0	1.00	26.55 ± 0.39	0	0	7	1.00	25.52 ± 0.59
2	0	0	0	1.00	44.82 ± 1.66	0	3	0	1.00	42.49 ± 1.13
3	0	0	0	1.00	46.39 ± 3.53	0	0	0	1.00	33.66 ± 0.89
4	0	0	2	1.00	51.92 ± 1.47	0	5	1	0.90	63.24 ± 7.17
5	0	0	4	1.00	35.14 ± 1.39	0	3	0	1.00	36.08 ± 2.56
6	0	0	0	1.00	23.32 ± 1.57	0	3	0	1.00	19.39 ± 0.60

**Table 8:** Moving obstacles scenario, low uncertainty. Risk-aware framework performance.

	Risk-Aware Framework			
Goal	Coll.	Aborts	Succ.	Time [s]
1	1	0	0.90	31.14 ± 1.79
2	0	0	1.00	43.30 ± 1.17
3	0	0	1.00	36.53 ± 1.89
4	2	0	0.80	45.23 ± 1.47
5	2	0	0.80	32.33 ± 1.87
6	0	0	1.00	19.31 ± 0.68

**Table 9:** Moving obstacles scenario, low uncertainty. Standard MPPI controller (without local path modification) with inflation radii 0.65 m and 0.85 m.

	Standard MPPI, Infl. 0.65 m				Standard MPPI, Infl. 0.85 m			
Goal	Coll.	Aborts	Succ.	Time [s]	Coll.	Aborts	Succ.	Time [s]
1	8	2	0.20	18.37 ± 1.85	3	1	0.60	20.26 ± 1.12
2	4	2	0.60	27.12 ± 0.25	2	4	0.80	27.32 ± 0.45
3	1	4	0.90	26.00 ± 0.65	7	1	0.20	26.57 ± 0.89
4	7	4	0.10	–	6	2	0.30	32.82 ± 1.10
5	6	3	0.40	19.79 ± 1.16	3	2	0.70	19.86 ± 0.34
6	2	1	0.80	15.99 ± 0.48	1	1	0.90	16.20 ± 0.52

**Table 10:** Cluttered and dynamic scenario, high uncertainty. Risk-aware framework versus standard MPPI with inflation radius 0.65 m.

	Risk-Aware Framework				Standard MPPI, Infl. 0.65 m			
Goal	Coll.	Aborts	Succ.	Time [s]	Coll.	Aborts	Succ.	Time [s]
1	1	0	0.90	32.01 ± 2.17	8	0	0.20	22.07 ± 0.87
2	3	0	0.70	43.99 ± 2.23	9	0	0.10	-
3	1	0	0.90	39.13 ± 3.44	3	0	0.70	34.52 ± 1.75
4	1	0	0.90	56.94 ± 1.66	4	1	0.60	48.42 ± 1.09
5	1	0	0.90	41.96 ± 5.95	4	0	0.60	25.21 ± 0.72
6	0	0	1.00	24.51 ± 1.52	6	0	0.40	18.40 ± 0.25

**Table 11:** Per-module CPU and memory footprint of the proposed framework in the cluttered scenario with dynamic obstacles and high uncertainty. CPU is expressed as a percentage of one logical core (100% = one core fully utilized). Values are the mean and the 95th percentile of the per-process CPU load over the full goal sequence after a 10 s warm-up; memory is the mean RSS over the same window. The framework total is obtained by summing the contributions of the individual modules.

Module	CPU Mean [%]	CPU $p_{95}$ [%]	RSS [MB]
Detection and tracking	14.5	18.4	31
Laser merger	6.4	8.0	14
Risk-map construction	37.6	54.0	28
Risk-area evaluation	22.0	24.0	41
Map fusion	6.1	8.0	11
Local A* path adaptation	44.6	80.0	82
Framework total	131.1	192.4	207

**Table 12:** Framework CPU usage across the three validation scenarios under the high-uncertainty configuration. Values are obtained by summing the per-module mean and 95th percentile CPU loads, expressed as a percentage of one logical core. The load increases monotonically with environmental complexity, dominated by the risk-map construction and the local A* path adaptation, whose work scales with the number of tracked obstacles.

Scenario	CPU Mean [%]	CPU $p_{95}$ [%]
Base (empty)	106.9	153.7
Cluttered (static)	123.9	189.0
Hybrid (static + dynamic)	131.1	192.4

## Data Availability

The implementation code is publicly available on GitHub at https://github.com/elestracca/local_risk_map_release (accessed on 15 June 2026). The parameters used are also provided in the Supplementary Materials.

## Supplementary Materials

The following supporting information can be downloaded at: https://www.mdpi.com/article/10.3390/s26123900/s1, Supplementary Material: simulation parameters. Table S1: Obstacle extractor parameters; Table S2: Circle tracker parameters; Table S3: Segment tracker parameters; Table S4: Actuation uncertainty estimator parameters; Table S5: Collision probability map parameters; Table S6: Risk planner parameters for the risk-aware framework; Table S7: MPPI controller parameters for the risk-aware framework; Table S8: MPPI controller parameters for the standard costmaps baseline; Table S9: Local path modification parameters for the standard costmap variant; Table S10: MPPI controller parameters for the standard costmaps with local path modification.
