# Supplementary material for: A Unified Local Risk Map for Uncertainty-Aware Mobile Robot Navigation in Cluttered and Dynamic Environments
Source: Sensors (Basel). 2026 Jun 19;26(12):3900. doi: 10.3390/s26123900 (PMC13307091; doi:10.3390/s26123900)
Supplement: Supplementary file 1 [file sensors-26-03900-s001.zip › sensors-4339751-supplementary.pdf]

Supplementary Material

A Unified Local Risk Map for Uncertainty-Aware Mobile Robot Navigation in Cluttered and Dynamic Environments

Elena Stracca, Olga Napolitano, Lucia Pallottino, Paolo Salaris

We list here all parameters used in the simulations presented in the main paper. Section 1 reports the parameters of the proposed risk-aware framework, organised by module: obstacle extraction (Table S1), circle tracking (Table S2), segment tracking (Table S3), actuation uncertainty estimation (Table S4), collision probability map (Table S5), risk planner (Table S6), and MPPI controller (Table S7). Section 2 reports the parameters of the standard MPPI baseline, and Section 3 those of the standard MPPI with local path modification.

1 Risk-Aware Framework Parameters

Obstacle extractor. Table S1 lists the segmentation and grouping thresholds used by the LiDAR point-cloud extractor to form circle and segment primitives from raw scans.

Table S1: Obstacle extractor parameters.

Parameter	Symbol	Value	Unit
Min. group points	–	5	–
Max. group distance	–	0.2	m
Max. split distance	–	0.08	m
Max. merge separation	–	0.3	m
Max. merge spread	–	0.3	m
Max. circle radius	–	0.6	m
Radius enlargement	–	0.0	m

Circle tracker. Table S2 reports the Kalman filter settings, track management rules, and dynamic/static classification thresholds for circle obstacles (Section 3.1.1 of the main paper).

Table S2: **Table S2:** Circle tracker parameters.

Parameter	Symbol	Value	Unit
Kalman filter			
Sensor rate	$1/\Delta t$	12.5	Hz
Process variance (static)	σ_a^2	0.01	$m^2\,s^{-3}$
Process variance (dynamic)	$\sigma_{a,\text{dyn}}^2$	0.05	$m^2\,s^{-3}$
Radius process noise	Q_r	$10^{-6}\,\Delta t$	m^2
Initial measurement variance	R_0	0.01	m^2
Initial velocity variance	–	0.1	$m^2\,s^{-2}$
Initial radius variance	–	0.03	m^2
EMA coefficient (position)	$\alpha_{R,x}{=}\alpha_{R,y}$	0.7	–
EMA coefficient (radius)	$\alpha_{R,r}$	0.5	–
Running-average EMA factor	–	0.3	–
Numerical floor	ϵ	10^{-4}	m^2
Fade variance rate	γ	0.002	m^2/frame
Track management			
Min. correspondence cost	–	0.6	m
Std. correspondence deviation	–	1.0	m
Tracking duration (fade)	–	1.0	s
Ring thickness (contour match)	–	0.05	m
Ring samples	–	48	–
Dynamic classification			
Mahalanobis threshold^2	–	4.0	–
Min. net displacement	–	0.1	m
Max. path/displacement ratio	–	1.5	–
Robot rotation suppression	–	0.3	rad/s
Dynamic promotion frames	–	2	frames
Static reversion threshold	–	0	frames
Close-range promotion distance	–	3.5	m

Segment tracker. Table S3 contains the data-association weights and gating parameters for segment obstacles (Section 3.1.2 of the main paper).

Table S3: Segment tracker parameters.

Parameter	Symbol	Value	Unit
Centre weight	w_c	1.0	–
Orthogonal weight	$w_\perp$	3.0	–
Angular weight	w_θ	0.5	m/rad
Gate scaling factor	k_{gate}	1.0	–
Association threshold	τ_{assoc}	0.6	m
Segment process variance	–	0.01	m^2

Actuation uncertainty estimator. Table S4 reports the parameters governing the sliding-window residual estimator described in Section 3.2 of the main paper, including the minimum variance floors that prevent overconfident estimates during low-excitation intervals.

Table S4: Actuation uncertainty estimator parameters.

Parameter	Symbol	Value	Unit
Sliding-window duration	–	3.0	s
Estimator rate	–	10	Hz
Lookahead distance	ℓ	0.5	m
Prediction horizon	τ	1.0	s
Bias weight	κ	0.5	–
Linear velocity gate	–	0.05	m/s
Angular velocity gate	–	0.10	rad/s
Min. σ_v floor	$\sigma_{v,\min}$	0.002	m/s
Min. σ_ω floor	$\sigma_{\omega,\min}$	0.005	rad/s
Covariance decay rate	–	0.2	1/s

Collision probability map. Table S5 collects the parameters of the probabilistic collision-risk map (Section 4), including the robot geometry, the two-pass evaluation thresholds, the dynamic-obstacle prediction settings, and the visibility mask configuration.

Table S5: Collision probability map parameters.

Parameter	Symbol	Value	Unit
Robot geometry			
Robot width	–	0.78	m
Robot height	–	0.98	m
Inscribed (collision) radius	r_{rob}	0.39	m
Circumscribed radius	r_{ext}	0.63	m
Map			
Local map dimension	–	3.5	m
Local map resolution	–	0.05	m
Two-pass evaluation			
Extended-radius scale factor	λ_{ext}	0.15	–
Upper probability cap	p_{hi}	0.10	–
Gating threshold	p_{lo}	0.002	–
Dynamic obstacles			
Prediction horizon	T_{hor}	3.5	s
Spatial step	δ_s	0.4	m
Reaction time	T_{react}	1.0	s
Max. robot speed	$v_{\max}$	1.0	m/s
Dynamic velocity threshold	–	0.2	m/s
Visibility			
Occlusion buffer radius	r_{occ}	0.7	m
Moving-segment clear radius	–	0.8	m
Risk weighting			
Risk weight (merge)	w_{risk}	0.8	–

Risk planner. Table S6 lists the grid resolution and map extent used by the A*-based local path modification module (Section 6.1).

Table S6: Risk planner parameters (risk-aware framework).

Parameter	Symbol	Value	Unit
Grid resolution	–	0.05	m
Extended map dimension	–	3.5	m

MPPI controller (risk-aware). Table S7 reports all MPPI parameters that differ from the Nav2 default configuration. The main differences with respect to the standard baseline include the use of a custom `ObstaclesRiskCritic` in place of the default `ObstaclesCritic`, and a local costmap composed of the `risk_layer` only, without the conventional obstacle and inflation layers.

Table S7: MPPI controller parameters (risk-aware framework). Only parameters differing from the Nav2 defaults are listed.

Parameter	Value	Nav2 def.	Unit
Trajectory sampling			
`time_steps`	30	56	–
`model_dt`	0.1	0.05	s
`batch_size`	1000	2000	–
`iteration_count`	2	1	–
`temperature`	0.1	0.3	–
`regenerate_noises`	true	false	–
`retry_attempt_limit`	5	1	–
`prune_distance`	3.0	1.7	m
`transform_tolerance`	0.2	0.1	s
Kinematic limits			
`vx_max`	1.0	0.5	m/s
`vx_min`	−0.5	−0.35	m/s
`vy_max`	0.0	0.5	m/s
`wz_max`	1.5	1.9	rad/s
Noise injection			
`vx_std`	0.5	0.2	m/s
`wz_std`	1.0	0.4	rad/s
Critics			
GoalCritic weight	6.0	5.0	–
PathAlignCritic weight	10.0	14.0	–
PathAlignCritic max occ. ratio	0.1	0.07	–
PathAlignCritic use orientations	true	false	–
PathAngleCritic weight	0.5	2.0	–
PathAngleCritic offset	20	4	–
Obstacle critic (custom ObstaclesRiskCritic)			
`repulsion_weight`	0.5	1.5	–
`critical_weight`	20.0	20.0	–
Local costmap (`risk_layer` only)			

2 Standard MPPI Baseline Parameters

The standard MPPI baseline uses the Nav2 default MPPI configuration with conventional obstacle and inflation costmap layers. Table S8 reports only the parameters that differ from the Nav2 defaults; all other settings retain their default values.

Table S8: MPPI controller parameters (standard costmaps baseline). Only parameters differing from the Nav2 defaults are listed.

Parameter	Value	Unit
Kinematic limits		
`vx_max`	1.0	m/s
`wz_max`	1.5	rad/s
Local costmap		
Plugins	`obstacle_layer + inflation_layer`	
`cost_scaling_factor`	3.0	–
`obstacle_max_range`	4.5	m
`raytrace_max_range`	7.0	m
Footprint	[±0.49, ±0.39] m (rectangular)	

3 Standard MPPI with Local Path Modification Parameters

This baseline augments the standard MPPI pipeline with the A*-based local path modification module described in Section 6.1, while retaining conventional costmap layers for the MPPI controller. Table S9 reports the planner-specific parameters, and Table S10 the MPPI settings. In the latter, a ✓ in the rightmost column marks parameters that also differ from the risk-aware configuration of Table S7.

Table S9: Local path modification parameters (standard costmap variant).

Parameter	Symbol	Value	Unit
Goal tolerance	–	0.3	m
Distance force accept	–	0.3	m
Cost risk weight	–	2.0	–
Improvement threshold (min)	–	0.0	–
Improvement threshold (max)	–	0.25	–
Path divergence d_0	–	0.15	m
Path divergence d_1	–	1.0	m
Path divergence γ	–	2.0	–
Grid resolution	–	0.05	m
Extended map dimension	–	3.5	m

Table S10: MPPI controller parameters (standard costmaps with local path modification). Only parameters differing from the Nav2 defaults are listed; ✓ marks those that also differ from the risk-aware framework (Table S7).

**Parameter**	**Value**	**Nav2 def.**	**Unit**	$\neq$ **Risk**
Trajectory sampling				
`time_steps`	30	56	–	
`model_dt`	0.1	0.05	s	
`batch_size`	1000	2000	–	
`iteration_count`	2	1	–	
`temperature`	0.1	0.3	–	
`regenerate_noises`	true	false	–	
`retry_attempt_limit`	5	1	–	
`prune_distance`	3.0	1.7	m	
`transform_tolerance`	0.2	0.1	s	
Kinematic limits				
`vx_max`	1.0	0.5	m/s	
`vx_min`	−0.5	−0.35	m/s	
`vy_max`	0.0	0.5	m/s	
`wz_max`	1.5	1.9	rad/s	
Noise injection				
`vx_std`	0.5	0.2	m/s	
`wz_std`	1.0	0.4	rad/s	
Critics				
GoalCritic weight	6.0	5.0	–	
PathAlignCritic weight	10.0	14.0	–	
PathAlignCritic max occ. ratio	0.1	0.07	–	
PathAlignCritic use orient.	true	false	–	
PathFollowCritic offset	40	30	–	
PathAngleCritic weight	0.5	2.0	–	
PathAngleCritic offset	20	4	–	
Obstacle critic (standard ObstaclesCritic)				
`repulsion_weight`	1.5	1.5	–	✓
`critical_weight`	20.0	20.0	–	
`consider_footprint`	true	true	–	✓
`collision_margin_dist.`	0.05	0.1	m	✓
`cost_scaling_factor`	3.0	3.0	–	✓
Local costmap				
Plugins	`risk_layer + obstacle + inflation`			✓
`cost_scaling_factor`	3.0	3.0	–	✓
`obstacle_max_range`	4.5	2.5	m	
`raytrace_max_range`	7.0	3.0	m	
